# Cost-effectiveness and budget impact of pembrolizumab+axitinib versus sunitinib in patients with advanced clear-cell renal cell carcinoma in the Netherlands

**DOI:** 10.3389/fonc.2023.1205700

**Published:** 2023-06-28

**Authors:** Nicolas S. H. Xander, W. Edward Fiets, Carin A. Uyl-de Groot

**Affiliations:** ^1^ Department of Health Technology Assessment, Erasmus School of Health Policy & Management, Erasmus University Rotterdam, Rotterdam, Netherlands; ^2^ Erasmus Centre for Health Economics Rotterdam, Erasmus University Rotterdam, Rotterdam, Netherlands; ^3^ Department of Medical Oncology, Medical Center Leeuwarden, Leeuwarden, Netherlands; ^4^ Institute for Medical Technology Assessment, Rotterdam, Netherlands

**Keywords:** cost-effectiveness, budget impact, pembrolizumab, axitinib, sunitinib, renal cell carcinoma, societal perspective

## Abstract

**Background:**

The phase 3 clinical trial KEYNOTE-426 suggested a higher efficacy regarding overall survival (OS) and progression-free survival (PFS) of pembrolizumab+axitinib compared to sunitinib as a first-line treatment for patients with advanced renal cell carcinoma. In this analysis, the potential cost-effectiveness of this combination treatment versus sunitinib for patients with advanced clear-cell renal cell carcinoma (accRCC) was examined from the societal perspective in the Netherlands.

**Methods:**

For this analysis, a partitioned survival model was constructed. Clinical data were obtained from the published KEYNOTE-426 trial reports; data on costs and (dis-)utilities were derived from published literature. Costs outside of the healthcare sector included treatment-related travel, informal care and productivity loss. Next to a probabilistic scenario analysis, various scenario analyses were performed that aimed at survival extrapolation, different utility values, treatment duration and drug pricing, as well as restricting the cohort to patients with an intermediate or poor prognosis. Further, a budget impact analysis over three years was conducted, in which a sensitivity analysis concerning ranges in costs and the number of patients was applied. Moreover, a scenario concerning increasing market penetration of pembrolizumab+axitinib up to a market share of 80% in the third year was analyzed.

**Results:**

The incremental cost-effectiveness ratio (ICER) of pembrolizumab+axitinib was estimated at €368,396/quality-adjusted life year (QALY) gained, with an incremental QALY gain of 0.55 over sunitinib. The probability of cost-effectiveness at a willingness-to-pay threshold of €80,000/QALY was estimated at 0%, a 50% probability was estimated at €340,000/QALY. Cost-effectiveness was not achieved in any of the applied scenarios. The budget impact over three years amounted to €417.3 million upon instantaneous and full replacement of sunitinib, and to €214.9 million with increasing market penetration.

**Conclusion:**

Pembrolizumab+axitinib was not estimated to be cost-effective compared to sunitinib as a first-line treatment for patients with accRCC in the Netherlands from a societal perspective. In none of the analyzed scenarios, cost-effectiveness was achieved. However, price reductions and shorter treatment durations might lead to a more favorable ICER.

## 1 Introduction

In 2020, 138,611 patients were diagnosed with kidney cancer across Europe (1). In the Netherlands, there were 2,697 diagnoses of kidney cancer in 2021, making it one of the 15 most common cancers nationwide (2, 3). Kidney cancer has an average five-year survival rate of approximately 67% across all age groups and disease stages (3). The most common type of kidney cancer (90% of all cases) is renal cell carcinoma (RCC) (4), of which 70% are clear-cell tumors (5). At diagnosis, about a third of cases are already metastatic; a further 20-50% of diagnosed patients will progress to that stage despite surgical treatment (6). At the advanced stage, which is characterized by the cancer having spread to other organs and/or distant lymph nodes (7), the five-year survival rate amounts to only 11% in the Netherlands (8).

In the past decade, treatment for advanced RCC (aRCC) has evolved considerably. Treatments targeting vascular endothelial growth factor (VEGF) have become prevalent, succeeding cytokine-based therapies (9). VEGF inhibitors rely on a VEGF blockade or on inhibiting VEGF receptors, or the signaling of the downstream receptors (10). A US-based study investigating the period from 2011 to 2015 found that 88% of aRCC patients received tyrosine kinase/VEGF-directed agents as treatment (11). At that time, the globally approved drug sunitinib was the standard treatment for patients with advanced clear-cell RCC (accRCC) (12). It is a tyrosine kinase inhibitor (TKI) targeting processes involved in tumor growth, progression, metastasis, and angiogenesis (13). According to a study by the Dutch Healthcare Institute (ZIN) in 2017, 73% of patients with RCC in the Netherlands received first-line systemic treatment with sunitinib (14).

More recently, immune checkpoint inhibiting agents have seen an increase of applications (9). Pembrolizumab is a humanized monoclonal IgG4 kappa anti-programmed cell death protein (PD1) antibody that inhibits cytotoxic activity upon being bound to the protein (15, 16). Axitinib is an oral second-generation TKI targeting VEGF receptors (16, 17). The KEYNOTE-426 study, a phase 3 randomized controlled trial (RCT), tested a combination treatment of pembrolizumab and axitinib as a first-line treatment for patients with accRCC (16, 18). Compared to sunitinib, the combination treatment showed a higher efficacy regarding overall survival (OS) and progression-free survival (PFS). Risk of death was estimated to be 47% lower (hazard ratio [HR] 0.53; 95% CI: 0.38–0.74) (16). The estimated median PFS at data cut-off was 15.1 months (95% CI: 12.6–17.7) for pembrolizumab+axitinib versus 11.1 months (95% CI: 8.7–12.5) for sunitinib across all IMDC risk categories (16). Median OS was not reached at data cut-off for pembrolizumab+axitinib (estimated OS rate at 24 months: 74.4% [95% CI: 69.9–78.2]) and estimated at 35.7 months for sunitinib (95% CI: 33.3–not reached) (18). In the follow-up publication, a median PFS of 15.4 months was reported for pembrolizumab+axitinib (95% CI: 12.7–18.9) compared to 11.1 months (95% CI: 9.1–12.5) for sunitinib (18). Based on the results of the KEYNOTE-426 trial, the European Medicines Agency (EMA) approved this new intervention as a first-line treatment for patients with aRCC (19).

While recommending the use of the combination treatment in the Netherlands, the Dutch Society for Medical Oncology (NVMO) voiced their concerns regarding the high costs of pembrolizumab+axitinib and the lack of information about the most efficient second-line treatment in case of progression (20). Whether this intervention is cost-effective for patients with accRCC in the Netherlands compared to sunitinib is not clear. Therefore, this study investigated whether pembrolizumab+axitinib would be cost-effective compared to sunitinib as a first-line treatment for patients with accRCC in the context of the Dutch health care system from a societal perspective.

## 2 Materials and methods

### 2.1 Model structure

**Figure 1:**
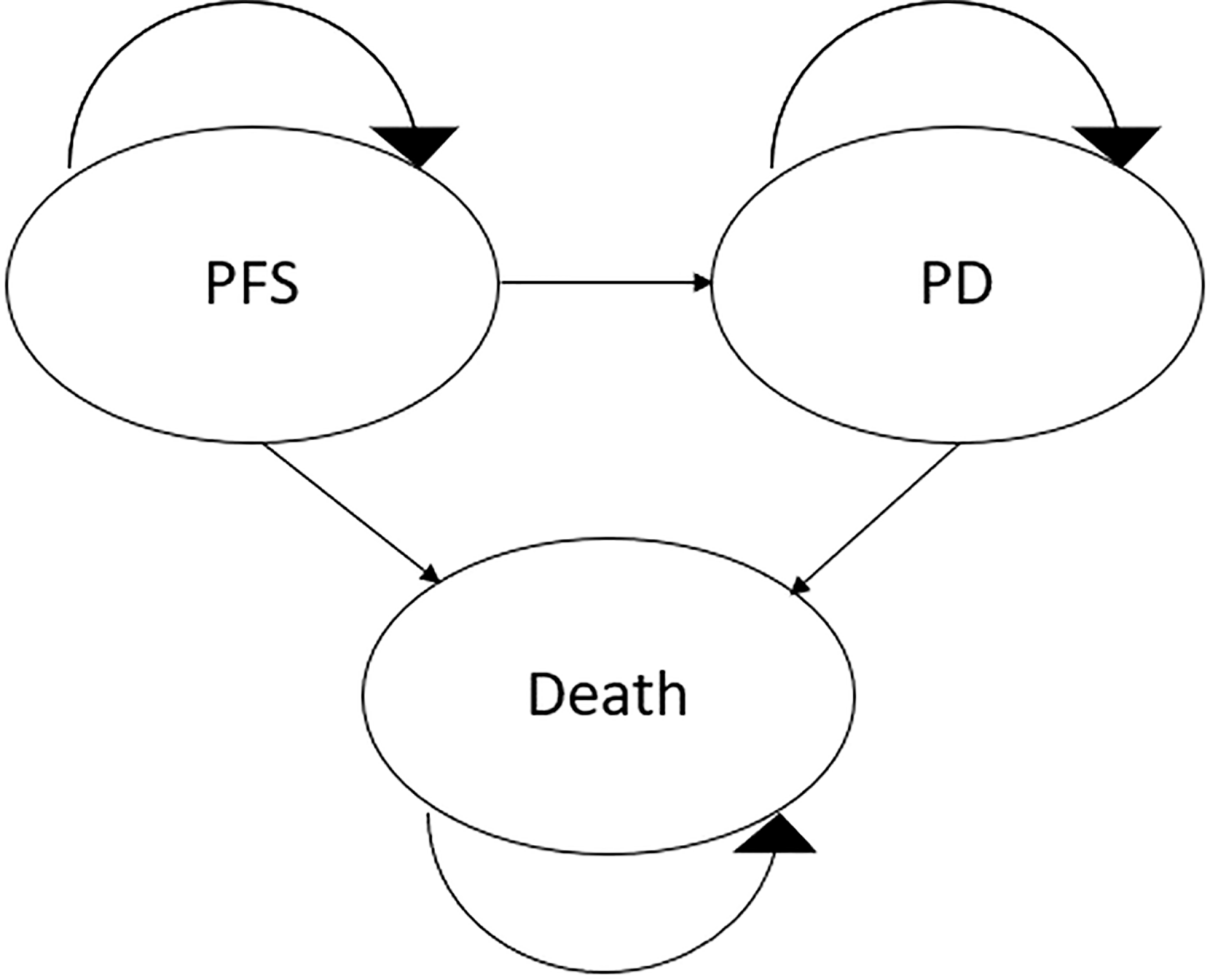
Diagrammatical representation of the model structure. PD, progressed disease; PFS, progression-free survival.

Following the modelling guidelines by the International Society for Pharmacoeconomics and Outcomes Research (ISPOR) (21–23), a partitioned survival model (PSM) based on a Markov decision model was constructed to estimate the treatment costs and effects of pembrolizumab+axitinib and sunitinib, respectively. This model included three different health states: PFS, progressed disease (PD), and death (**Figure 1**). For each treatment arm, the simulated cohort included 1,000 patients who were all assumed to meet the requirements for treatment, and to start treatment in the PFS state. Treatment with the intervention or the comparator would continue until disease progression or death, whichever occurred first. Upon entering the PD state, patients would either receive second-line chemotherapy or best supportive care. Discontinuation of treatment due to adverse events (AEs) was assumed and included in the model based on KEYNOTE-426 trial data (18) as follows: 21% of patients in the pembrolizumab+axitinib arm were assumed to permanently stop receiving pembrolizumab; 20% stopped being treated with axitinib. In the sunitinib arm, 12% of patients were assumed to discontinue treatment due to AEs (18). In both arms, discontinuation was modelled to occur after five cycles (15 weeks) based on corresponding data for the pembrolizumab+axitinib arm in the KEYNOTE-426 trial (16).

The model cycle length was three weeks. A time horizon of 15 years, tantamount to a lifetime horizon, was applied. Regarding the transition of patients to a different health state within a cycle, half-cycle correction was applied (23–25). The median age for each patient group – 62 years for pembrolizumab+axitinib, 61 years for sunitinib – was taken from the KEYNOTE-426 trial data (16, 18).

The primary model outputs were total costs, quality-adjusted life years (QALYs), and the incremental cost-effectiveness ratio (ICER). Life years (LYs) were used as an additional output. The primary data source for the model consisted of the results from the KEYNOTE-426 trial (16, 18). Further data were taken from publicly available databases (e.g., the Dutch Central Statistics Bureau [CBS]) and literature published in peer-reviewed journals. For the construction of the model, Microsoft Excel^®^ (Microsoft Corporation, Redmond, Washington, USA) was used.

### 2.2 Estimates on clinical outcomes and adverse events

For each treatment arm, parametric extrapolation curves were fitted to the Kaplan-Meier (KM) survival curves presented in the KEYNOTE-426 trial reports (16, 18). WebPlotDigitizer was used to obtain detailed values for time and PFS/OS probability throughout the clinical follow-up period. The obtained data at time points of every 1.5 months were applied to the curve-fitting method by Hoyle & Henley (26). The thus obtained data on progression and/or death and censorships served as input for a curve-fitting code compiled in RStudio. The gathered AIC scores and intercept and log(scale) values were included in the model to compute the extrapolation curves (exponential, Weibull, lognormal, loglogistic). For both treatment arms, the lognormal distribution was used to estimate the PFS, and the Weibull distribution to estimate the OS over the time horizon based on AIC score, face validity, and clinical plausibility (see **Supplementary Table 2** for the parameters, **Supplementary Figures 1**–**4** for the extrapolated survival curves) (27). Clinical plausibility of the extrapolation was further tested against the PFS and OS rates in the KEYNOTE-426 trial at the 42-month follow-up point (28). Moreover, assuming better long-term OS for patients treated with pembrolizumab+axitinib, survival rates for sunitinib were modelled not to surpass the rates for the combination treatment at any point within the time horizon.

Background mortality was accounted for to adjust the probability of transitioning to the death state at different ages. For the respective median ages of each treatment arm, the rate of death was calculated based on age-stratified instantaneous mortality data as well as the follow-up life years within the time horizon. Mortality data was taken from life tables for 2020 published by CBS (29). To avoid double-counting, the background mortality coefficient was adjusted for deaths by ccRCC with data taken from IKNL and published literature (**Supplementary Table 4**) (3–5).

Grade 3/4 AE with an incidence of ≥5% were included in the model (18). In order to avoid double-counting regarding costs and dis-utilities, thrombocytopenia was assumed to be the same as decreased platelet count. The same assumption applied to neutropenia and decreased neutrophil count. The prevalence of AEs to estimate the patient-based dis-utilities and costs was taken from the KEYNOTE-426 trial reports (18).

### 2.3 Utility and cost estimates

QALYs were estimated by adjusting the gained LYs by health-related quality of life (HRQoL). Utility-related inputs based on EQ-5D data (30) were derived from literature. The baseline utility as well as utility with the pembrolizumab+axitinib treatment was assumed at 0.76, with the sunitinib treatment at 0.72; the utility in the PD state was assumed at 0.66 for all patients (31, 32).

For AE-related dis-utilities, estimates were taken from published literature as well as from publications by ZIN on comparable treatments for the same indication (14, 33–35). Where dis-utilities for AE were not available for accRCC patients, corresponding values for comparable indications were utilized. Details are shown in **Table 1** and **Supplementary Table 1**. Differing utility values applied in published cost-effectiveness analyses (CEAs) on pembrolizumab+axitinib versus sunitinib were included as scenarios in the sensitivity analysis (47, 48).

**Table 1:** Model input parameters (baseline values, ranges, distributions for sensitivity analysis).

Variable	Baseline value (95% CI)	Reference	Distribution
Treatment-based health state utility parameters			
**Baseline utility**	0.760 (0.700–0.820)	(31)	beta
**Utility PFS P+A**	0.760 (0.710–0.810)	(31)	beta
**Utility PFS sunitinib**	0.720 (0.649–0.791)	(32)	beta
**Utility PD**	0.660 (0.547–0.773)	(31)	beta
Cost parameters for drug acquisition in PFS and treatment administration			
**Price of pembrolizumab (50 mg)**	€1,430.28	(36)	gamma
**Price of axitinib (per 5 mg)**	€68.78	(37)	gamma
**Price of sunitinib (per 50 mg)**	€113.93	(38)	gamma
**Administration costs pembrolizumab**	€308.98 (€248.42–€369.55)	(14, 39)	gamma
**Pharmaceutical delivery costs per pick-up of axitinib/sunitinib (indexed)**	€6.62	(40)	gamma
Healthcare resource use cost parameters in PFS			
**Visit to outpatient clinic**	€100.00 (€80.40–€119.60)	(41)	gamma
**Inpatient care**	€537.00 (€431.75–€642.25)	(41)	gamma
**Daycare treatment**	€308.98 (€248.42–€369.55)	(41)	gamma
**Emergency treatment**	€290.26 (€233.37–€347.15)	(14)	gamma
**Blood test (lab)**	€12.48 (€10.04–€14.93)	(14)	gamma
**X-ray**	€58.26 (€46.84–€69.68)	(14)	gamma
**CT scan**	€209.11 (€168.12–€250.10)	(14)	gamma
**MRI scan**	€376.61 (€302.79–€450.42)	(14)	gamma
**Ultrasound**	€125.88 (€101.21–€150.56)	(14)	gamma
**EKG**	€50.98 (€40.99–€60.97)	(14)	gamma
Patient distribution regarding second-line treatment after disease progression (first-line treatment P+A)			
**Best supportive care only**	50.00%	(16)	Dirichlet
**Cabozantinib**	30.00%	expert opinion	Dirichlet
**Nivolumab**	15.00%	expert opinion	Dirichlet
**Everolimus**	1.00%	expert opinion	Dirichlet
**Axitinib**	2.50%	expert opinion	Dirichlet
**Sunitinib**	1.50%	expert opinion	Dirichlet
Patient distribution regarding second-line treatment after disease progression (first-line treatment sunitinib)			
**BSC only**	39.30%	(16)	Dirichlet
**Cabozantinib**	12.14%	expert opinion	Dirichlet
**Nivolumab**	48.56%	expert opinion	Dirichlet
**Everolimus**	0.00%	expert opinion	Dirichlet
**Axitinib**	0.00%	expert opinion	Dirichlet
**Sunitinib**	0.00%	expert opinion	Dirichlet
Cost parameters for travel and informal care			
**Cost per km travelled**	€0.30	(42)	gamma
**Distance to hospital [km]**	7.0 (4.26–9.74)	(40)	gamma
**Distance to pharmacy [km]**	1.3 (0.79–1.81)	(40)	gamma
**Informal care costs per hour (indexed)**	€15.55 (€12.50–€18.60)	(40)	gamma
Input parameters regarding productivity loss			
**Friction period [days]**	138.439	(40, 43, 44)	gamma
**Productivity cost per hour**	€37.30 (€22.68–€51.92)	(44)	gamma
**Average working hours per week**	32.1 (19.517–44.683)	(45)	gamma
**Share of working patients in the PFS state**	20% (12.160%–27.840%)	(14, 39, 44)	gamma
**Share of working patients in the PD state**	0%	(14, 39, 44)	
**Hours of unpaid work lost due to treatment administration at hospital**	0.38 (0.104–0.656)	(46)	gamma
**Hours of unpaid work lost due to treatment administration at home**	0.41 (~0–0.843)	(46)	gamma
**Cost unpaid work per hour [indexed]**	€15.55 (€9.45–€21.64)	(40)	gamma

BSC, best supportive care; CI, confidence interval; CT, computer tomography; EKG, electrocardiogram; MRI, magnet resonance imaging; P+A, pembrolizumab+axitinib; PD, progressed disease; PFS, progression-free survival. Costs are indexed to 2021.

In accordance with the societal perspective chosen for this analysis, healthcare and societal costs were considered. Healthcare-related costs included costs for drug acquisition, administration, AE treatment, healthcare resource use, best supportive care, and end-of-life care. Costs outside of healthcare included travel costs, informal care costs, and productivity loss (**Table 1**; **Supplementary Table 1**). All costs were inflation-adjusted to 2021 values in euros with the Dutch Consumer Price Index as reference (40, 49). Drug acquisition and administration data as foundation for the corresponding costs were based on the KEYNOTE-426 trial (16, 18). Pembrolizumab was administered intravenously at a 200 mg dose once every three weeks for a maximum of 35 cycles (barring earlier disease progression or death). Axitinib was administered orally at a dose of 5 mg twice daily; treatment routine was assumed to last until disease progression (16, 18); Sunitinib was administered orally at 50 mg once a day for the first four weeks of a six-week cycle (16, 18). As with axitinib, treatment was assumed to continue until disease progression (18). To fit the three-week cycle applied in the model, administration of sunitinib was assumed to occur for 14 out of 21 days. Costs for AE treatment were derived from published literature (50–52).

Regarding treatment upon disease progression, 50% of patients in the pembrolizumab+axitinib arm and 60.7% in the sunitinib arm were assumed to receive second-line treatment (16). The distributions regarding these treatments were based on relevant results of the KEYNOTE-426 trial (16), as well as a Dutch expert estimate, and are shown in **Table 1** and **Supplementary Table 1**. The treatment durations were taken from the recommendation by ZIN regarding avelumab+axitinib for accRCC (14).

Travel costs incurred for receiving intravenous (IV) chemotherapy and for healthcare resource use at the hospitals, as well as for receiving orally administered drugs (self-administration assumed) at the pharmacy. Costs for informal care were based on the proxy good method (40, 53); published recommendations by ZIN on aRCC treatments served as reference for the hours spent on such care by an informal caregiver (**Table 1**; **Supplementary Table 1**) (14, 39). Costs regarding absenteeism-related productivity loss were estimated through applying the friction cost method and using relevant data from CBS (30, 43–45). 20% of patients were assumed to still be working in the PFS state, with the remainder already being retired (14, 39). In the PD state, all patients were assumed to have retired. Further, productivity losses for unpaid work on part of the patient and of their informal caregiver during the friction cost period (40) were included. Such losses were assumed to be linked to first- and second-line treatment administrations (46). The costs were based on hourly proxy costs for cleaning labor in the Netherlands (40). On part of the informal caregiver, working hours lost included absenteeism and presenteeism (54). A 40% increase in working hours lost was assumed for informal caregivers of patients in the PD state (14, 39).

In accordance with the ZIN reference case, an annual constant discount rate of 4% for costs and 1.5% for effects was adhered to (30). The willingness-to-pay (WTP) threshold was set at €80,000/QALY as the applicable threshold for aRCC in the Netherlands (14, 55).

### 2.4 Sensitivity analysis

A probabilistic sensitivity analysis (PSA) was conducted to illustrate the robustness of our results. 1,000 Monte Carlo simulations were conducted; for parameters constrained between 0 and 1 (utility values and AE incidences), beta distributions were used, and gamma distributions were applied to costs and other parameters constrained to values >0 (56, 57). Multinominal data regarding subgroups of patients in the PD state was subject to a Dirichlet distribution (56). Uncertainty regarding survival data was accounted for by applying variance-covariance matrices using the Cholesky decomposition method in the PSA (56). Where possible, 95% confidence intervals (CI) were determined through applying the input parameter’s standard error (SE) values. Subsidiarily, the SE was assumed as a fraction of the parameter’s mean value. A detailed schedule of the parameters and how the probabilistic values were determined is provided in **Supplementary Table 3**. A cost-effectiveness plane was constructed to show the base-case ICER and the uncertainty surrounding costs and effects. Cost-effectiveness acceptability curves (CEACs) were designed to determine the cost-effective intervention (57).

Moreover, several scenario analyses were carried out. These involved alternative distributions for PFS and OS, best-/worst-case scenarios regarding utility values, and utility values based on published CEAs on pembrolizumab+axitinib versus sunitinib (47, 48). Regarding cost parameters, hypothetical lower pricings of pembrolizumab and axitinib (decrease by 10%, 20%, and 50%, respectively) were considered. Further, a shortened first-line treatment duration of 35 and 15 cycles, respectively, was assumed. A different distribution of second-line treatment drugs based on an expert report referenced in a recommendation by ZIN constituted a further scenario. A further scenario applied a second-line treatment patient distribution in accordance with an expert estimate referenced in the ZIN recommendation on avelumab+axitinib (**Table 1**; **Supplementary Table 1**) (14). An additional scenario exclusively included patients with an intermediate/poor IMDC risk score. Sub-scenarios to this scenario concerned price reductions and shortened first-line treatment durations as mentioned above. The relevant survival probabilities were modelled after the specific KM curves in the KEYNOTE-426 trial (18), with the same selection process for the curve-fitting distribution being applied. For all scenario analyses, all parameters that were not altered in view of the respective scenario were held constant.

### 2.5 Budget impact analysis

Based on the base case results of the CEA, a budget impact analysis (BIA) was conducted from the national-governmental perspective of the Dutch government as the budget holder. Following the ZIN reference case, the analysis included healthcare-related costs over a time horizon of three years (30). Costs were not discounted (58). The patient population was, as a first step, derived from kidney cancer incidence figures for 2019 through 2021 (3). In order to estimate the yearly number of new patients with accRCC, the respective shares of patients with RCC (90%) and ccRCC (70% of RCC patients) were taken from existing studies (4, 5). Further literature was utilized to account for patients whose disease has already advanced at the time of diagnosis (33%) and, respectively, would progress to the advanced stage despite surgical resection (35% as mean value of 20%–50%) (6). The share of accRCC patients eligible for the treatment was derived from the KEYNOTE-426 trial reports (16, 18). No transfer of patients from the standard treatment to the alternative treatment was assumed. To perform the analysis, the BIA calculation tool by The Netherlands Organization for Health Research and Development (ZonMw) was utilized (59). Three scenarios were considered to account for the uncertainty of the cost parameters. The main scenario was built on the base case results of the CEA, the second scenario concerned a 15-cycle treatment for both treatment arms and prices for pembrolizumab and axitinib reduced by 50%, and the third scenario exclusively involved patients with an intermediate/poor IMDC risk score. For this particular scenario, patient data regarding that risk group were obtained from a real-world study on aRCC patients in the Netherlands (60).

A sensitivity analysis was performed to account for the uncertainty regarding patient and cost data. In accordance with the calculation tool’s capacities, ranges of 90%–115% for the patient population and of 80%–130% for costs were assumed. Moreover, given that the base case of the BIA assumed an instantaneous and full replacement of sunitinib by pembrolizumab+axitinib on the market, a scenario analysis was included that assumed a market penetration of the combination treatment of 25% in the first year, 50% in the second year, and 80% in the third year.

## 3 Results

### 3.1 Base case

The extrapolated survival curves derived from the KM curves for OS and PFS in the clinical trial are shown in **Supplementary Figures 1**–**4**. For pembrolizumab+axitinib, the median PFS rate was estimated at approximately 15.5 months, compared to approximately 9.8 months for sunitinib. According to the extrapolation in the model that was adjusted for background mortality, the median OS rate was estimated at 42.9 months for pembrolizumab+axitinib and 31.9 months for sunitinib. 33.7% of patients treated with pembrolizumab+axitinib were projected to reach 5-year survival compared to 25.6% of patients receiving treatment with sunitinib.

Compared with patients treated with sunitinib, the model projected a gain of 0.55 QALYs (2.79 versus 2.25 QALYs) and 0.65 LYs (4.02 versus 3.37 LYs) for patients receiving pembrolizumab+axitinib over a 15-year time horizon. Detailed cost results are shown in **Table 2**.

**Table 2:** Base case results.

Cost element	Pembrolizumab+axitinib	Sunitinib	Increment
PFS state			
**Drug acquisition**	€ 194,055	€ 36,633	€ 157,423
**Treatment administration**	€ 5,689	€ 80	€ 5,610
**Healthcare resource use**	€ 47,861	€ 18,849	€ 29,011
**AE treatment**	€ 630	€ 751	-€ 121
**Travel**	€ 913	€ 561	€ 352
**Informal care**	€ 10,809	€ 9,528	€ 1,281
**Productivity loss (absenteeism)**	€ 18,944	€ 18,944	€ 0
**Productivity loss unpaid work**	€ 8,608	€ 2,050	€ 6,558
**Productivity loss informal caregiver**	€ 23,657	€ 16,413	€ 7,244
**costs healthcare perspective PFS**	€248,236	€56,313	€191,722
**costs societal perspective PFS**	€311,172	€104,016	€207,156
PD state			
**Drug acquisition**	€ 21,003	€ 30,239	-€ 9,236
**Treatment administration**	€ 857	€ 2,891	-€ 2,034
**Healthcare resource use**	€ 21,583	€ 21,409	€ 174
**Best supportive care**	€ 1,781	€ 1,483	€ 298
**End-of-life costs**	€ 934	€ 1,950	-€ 1,016
**Travel costs**	€ 12,420	€ 12,760	-€ 340
**Informal care**	€ 12,155	€ 13,444	-€ 1,289
**Productivity loss (absenteeism)**	€ 4,094	€ 4,337	-€ 243
**Productivity loss unpaid work**	€ 14,098	€ 6,912	€ 7,187
**Productivity loss informal caregiver**	€ 980	€ 1,038	-€ 58
**costs healthcare perspective PD**	€57,645	€68,783	-€11,138
**costs societal perspective PD**	€89,906	€96,463	-€6,557
*Total costs*			
**Total costs healthcare perspective**	€305,881	€125,096	€180,785
**Total costs societal perspective**	€401,071	€200,272	€200,799

AE, adverse events; PD, progressed disease; PFS, progression-free survival.

The total costs for the pembrolizumab+axitinib treatment amounted to €401,071 versus €200,272 for sunitinib, resulting in an increment of €200,799. Acquisition costs in the PFS state were estimated at €194,055 for pembrolizumab+axitinib and at €36,633 for sunitinib. Administration costs in the PFS state amounted to €5,689 for pembrolizumab+axitinib and €80 for sunitinib. Costs for the use of healthcare resources in the PFS state were estimated at €47,861 for pembrolizumab+axitinib and at €18,849 for sunitinib; in the PD state, these costs amounted to €21,583 in the pembrolizumab+axitinib group and to €21,409 in the sunitinib group. Costs outside of the healthcare sector (such as travel costs, costs for informal care, productivity loss) were estimated at €62,930 (pembrolizumab+axitinib) versus €47,496 (sunitinib) in the PFS state and at €32,261 (pembrolizumab+axitinib) versus €27,680 (sunitinib) in the PD state, respectively.

The resulting ICER was €368,396 per QALY gained, which was higher than the set WTP threshold of €80,000/QALY. Costs only concerning the healthcare perspective amounted to €305,881 for pembrolizumab+axitinib and €125,096 for sunitinib, resulting in an increment of €180,785 and an ICER of €331,677.

### 3.2 Sensitivity analysis

**Figure 2:**
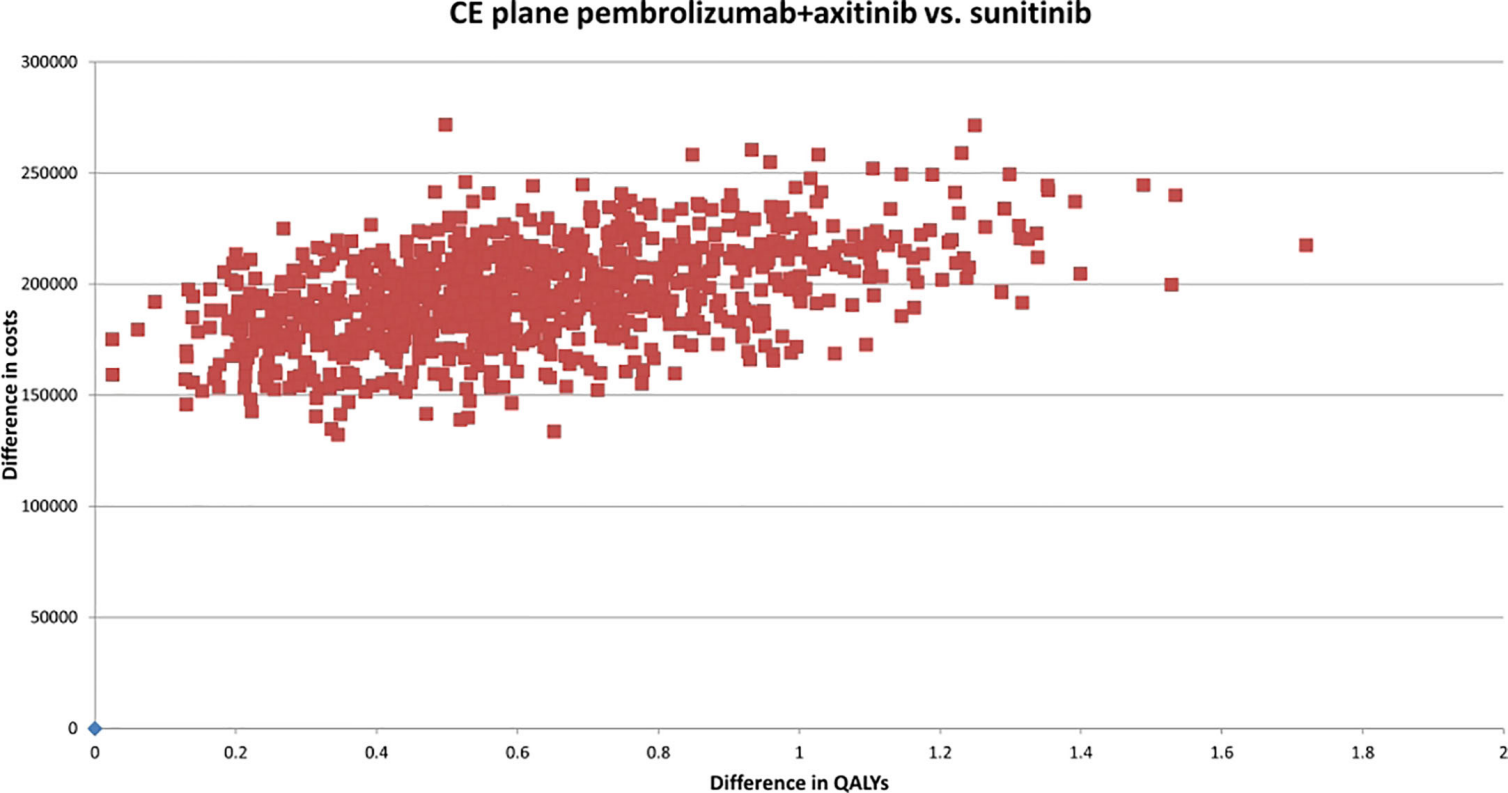
Cost-effectiveness plane of pembrolizumab+axitinib versus sunitinib. CE, cost-effectiveness; QALYs, quality-adjusted life years; vs., versus.

**Figure 3:**
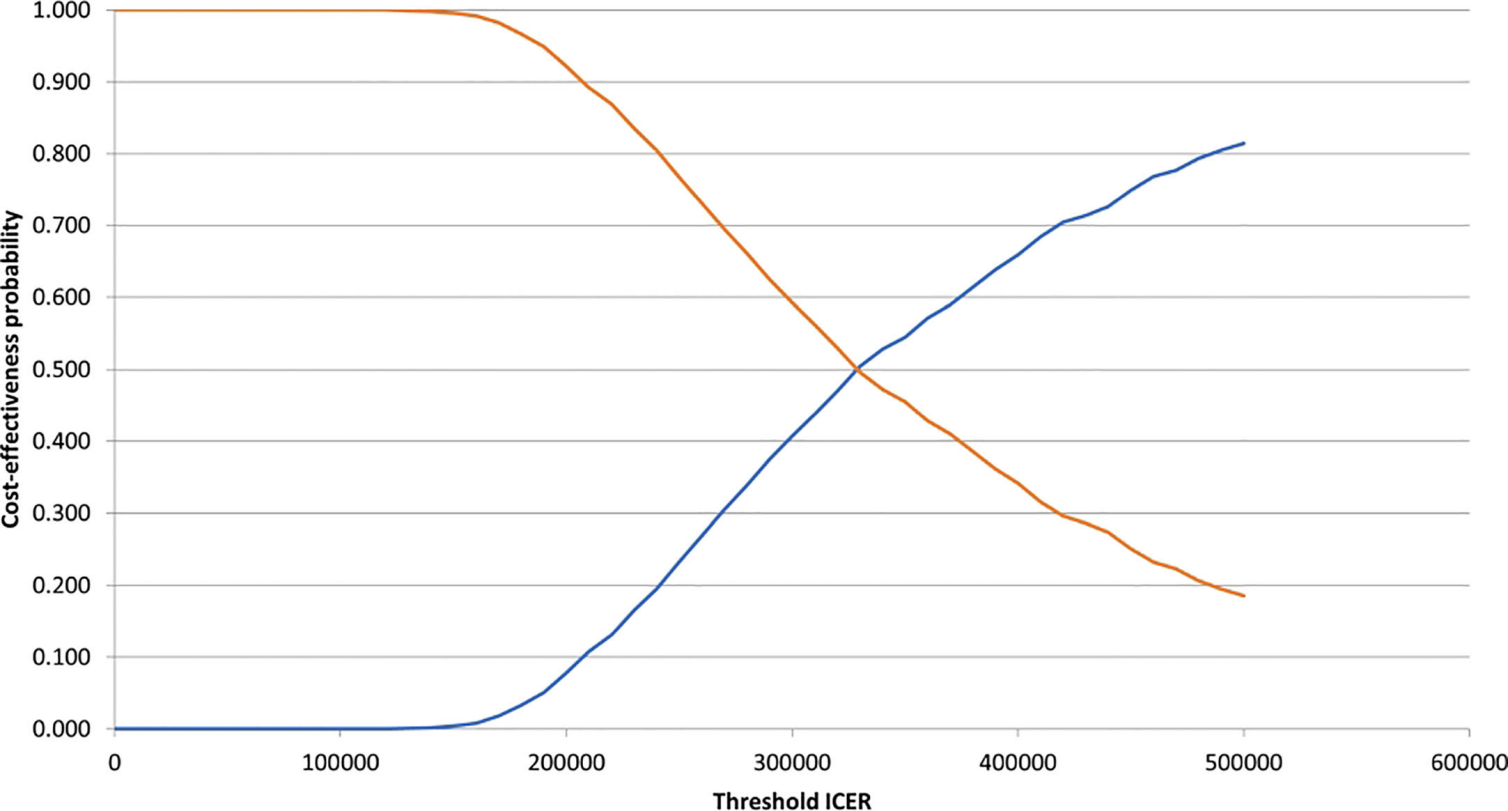
Cost-effectiveness acceptability curves for pembrolizumab+axitinib and sunitinib. ICER, incremental cost-effectiveness ratio. blue line, pembrolizumab+axitinib; orange line, sunitinib.

According to the constructed cost-effectiveness plane (**Figure 2**), the PSA suggested upon visual inspection that the pembrolizumab+axitinib treatment was clinically more effective, but also more costly than sunitinib since all analysis iterations were in the north-eastern quadrant. The cost-effectiveness probability at the selected WTP threshold was 0%, suggesting that the combination treatment was not cost-effective compared to sunitinib. The point of intersection of the CEACs for each treatment arm, indicating an equal probability of acceptability (i.e., 50%), was estimated at approx. €340,000 (**Figure 3**), which supported the suggested lack of cost-effectiveness.

### 3.3 Scenario analyses

**Table 3** summarizes the results of the scenario analyses. In none of the scenarios, cost-effectiveness under the WTP threshold of €80,000/QALY was achieved. Reducing the price of pembrolizumab and axitinib by 50% lowered the ICER to; restricting the treatment duration in the PFS state to 15 cycles decreased it further to €132,637/QALY. When only including patients with an intermediate or poor IMDC risk score in the population, the ICER slightly decreased to €357,988/QALY. None of the applied sub-scenarios showed pembrolizumab+axitinib as cost-effective. However, reducing the treatment duration to 15 cycles and the costs for pembrolizumab+axitinib by 50% decreased the ICER by more than two thirds to €118,343.

**Table 3:** Scenario analysis results.

Scenario	Total cost (P+A)	Incremental cost	Total effects (QALY, P+A)	Incremental effects	ICER
***Base case***	*€401,071*	*€200,799*	*2.79*	*0.55*	*€368,396*
Distribution-related scenarios					
**OS: Weibull; PFS: Weibull**	€388,455	€195,186	2.75	0.52	€372,677
**OS: exponential; PFS: lognormal**	€429,985	€226,297	3.46	1.11	€203,709
**OS: exponential; PFS: Weibull**	€413,401	€215,029	3.41	1.07	€200,076
**OS: loglogistic; PFS: lognormal**	€429,951	€210,662	3.47	0.62	€340,797
**OS: loglogistic; PFS: Weibull**	€413,055	€200,089	3.41	0.58	€343,697
Utility-based scenarios					
**Base case utilities: best case**	€401,871	€200,799	3.10	0.53	€375,609
**Base case utilities: worst case**	€401,871	€200,799	2.49	0.56	€361,455
**Utility PFS P+A: 0.82; Sun: 0.73** (48)	€401,871	€200,799	2.93	0.67	€299,130
**Utility PFS P+A: 0.76; Sun: 0.73** (47)	€401,871	€200,799	2.79	0.53	€378,996
Cost-related scenarios					
**10% decrease of pembrolizumab+axitinib price**	€381,600	€181,328	2.79	0.55	€332,673
**20% decrease of pembrolizumab+axitinib price**	€362,129	€161,857	2.79	0.55	€296,950
**50% decrease of pembrolizumab+axitinib price**	€303,715	€103,443	2.79	0.55	€189,782
**Base case price, 35-cycle treatment duration for all drugs in PFS state**	€356,083	€168,805	2.79	0.55	€309,698
**Base case price, 15-cycle treatment duration for all drugs in PFS state** (20)	€287,391	€109,634	2.79	0.55	€201,140
**50% decrease of pembrolizumab+axitinib price, 15-cycle treatment duration for all drugs**	€242,673	€64,916	2.79	0.55	€119,098
**Patient distribution for second-line treatment as per ZIN recommendation on avelumab+axitinib** (14)	€403,354	€201,243	2.79	0.55	€369,211
Survival-related scenario and sub-scenarios					
**Based on survival curves with intermediate/poor IMDC prognosis** (18)	€372,291	€185,379	2.46	0.52	€357,688
**Distribution: OS & PFS, Weibull**	€363,330	€183,825	2.43	0.50	€360,901
**50% decrease of pembrolizumab+axitinib price**	€281,922	€95,010	2.46	0.52	€183,321
**Base case price, 15-cycle treatment duration for all drugs in PFS state** (20)	€271,345	€104,944	2.46	0.52	€202,489
**50% decrease of pembrolizumab+axitinib price, 15-cycle treatment duration for all drugs**	€227,734	€61,334	2.46	0.52	€118,343

ICER, incremental cost-effectiveness ratio; IMDC, International Metastatic Renal Cell Carcinoma Database Consortium; OS, overall survival; PFS, progression-free survival; P+A, pembrolizumab+axitinib; QALY, quality-adjusted life year; Sun, sunitinib.

### 3.4 Budget impact analysis

Based on the relevant patient data for 2019–2021, and accounting for the share of accRCC patients eligible for treatment, the number of patients in the base case scenario as well as in the first alternative scenario (price reduction of 50% for pembrolizumab+axitinib, 15 treatment cycles for both treatment arms) was estimated at 798 in the first year, 729 in the second year, and 781 in the third year. In the second alternative scenario (only patients with an intermediate or poor IMDC risk score), we estimated the population of new patients per year at 91% of the base case population, thus at 726, 663, and 711, respectively. At the base case, the healthcare-related costs per patient amounted to €305,881 (pembrolizumab+axitinib) versus €125,096 (sunitinib), resulting in an increment of €180,785. The expenses per patient in the first alternative scenario were estimated at €153,537 for pembrolizumab+axitinib and €103,823 for sunitinib, respectively, with an increment of €49,713. In the second alternative scenario, costs in the pembrolizumab+axitinib arm were estimated at €284,720, and at €117,610 for the sunitinib arm, resulting in an increment of €167,110. the total budget impact over three years amounted to €417.3 million at the base case. Applying 15 treatment cycles to both treatment arms in the first line and decreasing the prices of pembrolizumab and axitinib by 50% lowered the total impact to €114.8 million. Limiting the patient population to patients with an intermediate or poor IMDC risk score decreased the impact to €350.9 million. Based on the ranges applied in the sensitivity analysis, the total budget impact ranged from €300.4 million to €624.0 million at the base case. The scenario related to treatment duration and costs resulted in a budget impact range of €82.7 million to €171.6 million, and the impact for the scenario regarding patients with an intermediate or poor IMDC risk score ranged from €252.9 million to €524.7 million. The results of the analysis are summarized in **Table 4**.

**Table 4:** Budget impact analysis results.

Scenario	2019 data	2020 data	2021 data	Total
Population of eligible patients				
Base case scenario	798	729	781	2,308
1^st^ alternative scenario (decreased drug prices, 15 treatment cycles)	798	729	781	2,308
2^nd^ alternative scenario (only patients with intermediate/poor IMDC risk prognosis)	726	663	711	2,100
Budget impact (× €1,000)				
Base case scenario	€144,266	€131,792	€141,193	€417,252
1^st^ alternative scenario	€39,671	€36,241	€38,826	€114,739
2^nd^ alternative scenario	€121,322	€110,794	€118,815	€350,931
Budget impact range (× €1,000)				
Base case scenario	€103,843 – €215,749	€94,876 – €196,947	€101,673 – €211,283	€300,392 – €623,979
1^st^ alternative scenario	€28,555 – €59,328	€26,090 – €54,158	€27,959 – €58,100	€82,604 – €171,586
2^nd^ alternative scenario	€87,432 – €181,398	€79,812 – €165,539	€85,560 – €177,705	€252,804 – €524,642
Budget impact (× €1,000) with assumed increasing market penetration of P+A				
Market share	25%	50%	80%	
Base case scenario	€36,073	€65,861	€113,003	€214,936
1^st^ alternative scenario	€9,920	€18,111	€31,074	€59,105
2^nd^ alternative scenario	€30,343	€55,400	€95,054	€180,797

IMDC, International Metastatic Renal Cell Carcinoma Database Consortium.

Accounting for increasing market penetration changed the total budget impacts to €214.9 million (base case), €59.1 million (15 treatment cycles, 50% cost reduction for both pembrolizumab and axitinib), and €180.8 million (intermediate/poor IMDC risk score).

## 4 Discussion

We conducted a CEA and BIA of pembrolizumab+axitinib compared to sunitinib as a first-line treatment for accRCC from a societal perspective. To our knowledge, this is the first analysis comparing these two treatments from this perspective using Dutch data for costs in particular. Based on our model, the ICER for pembrolizumab+axitinib amounted to €368,396/QALY gained. The PSA resulted in a 0% WTP probability at a threshold of €80,000/QALY. At an ICER of approximately €340,000/QALY gained, that probability increased to 50%. This suggests that pembrolizumab+axitinib is not cost-effective compared to sunitinib for the relevant indication at the applied threshold. The estimated 3-year budget impact at the base case amounted to €417.3 million (€214.9 million when accounting for increasing market penetration) with a range of €300.4 million to €624.0 million.

Previously published CEAs on this intervention as first-line treatment for accRCC were conducted from the US healthcare (payer) perspective (47, 48, 61) as well as from the healthcare perspective in China (62). Compared to those studies, the QALY increment in our analysis was substantially lower. For instance, the increment in the analysis by Ding et al. was estimated at 1.18 QALYs (47); in the analysis by Bensimon et al., an increment of 2.72 QALYs was reported (61); Zhu et al. estimated the incremental QALYs at 1.61 (48).This discrepancy might be explained by different modelling approaches regarding survival extrapolation and highly influenced the difference in the ICER and the overall conclusion. The restriction to healthcare-related costs and different pricing schemes in the US and in China might also have factored into the different estimates.

We observed that the expensive pricing of pembrolizumab and its IV administration, and the overall costs of the combination treatment were the most prominent factors in influencing the ICER. This was supported by the scenario analysis since decreasing the acquisition costs for both drugs of the combination treatment lowered the ICER to a considerable extent, albeit unable for pembrolizumab+axitinib to achieve cost-effectiveness of its own. Further, the incremental healthcare resource use costs, which were estimated at €29,011, significantly contributed to the presented ICER as well.

Given the estimated ICER in view of the large cost increment and the relatively low increment in QALYs, achieving cost-effectiveness of the pembrolizumab+axitinib treatment compared to sunitinib might, in our opinion, be regarded as unlikely. However, combining possible approaches might still yield promising outcomes. A case could be made for a combined price reduction of both pembrolizumab and axitinib for this specific indication as one of the necessary steps towards cost-effectiveness, since a reduction by 50% essentially halved the ICER in our model. Moreover, given the large number of indications for which pembrolizumab is used (63), adapted pricing arrangements tailored to specific indications like a(cc)RCC, or managed entry agreements could be negotiated. Furthermore, earlier discontinuation of treatment with pembrolizumab (as currently researched with a different indication in the Safe Stop trial (64)) could, in case of an at least comparable outcome on survival and HRQoL and subsequent implementation in practice, further reduce drug acquisition and administration costs for the combination treatment. Restricting the utilization of the combination treatment to patients with an intermediate or poor prognosis according to the IMDC risk score might constitute a further option, although the estimated effect on the ICER compared to the base case, which includes patients of all risk groups, might only be modest. However, even combining both approaches are still unlikely to lead to a cost-effective outcome under the used WTP threshold of €80,000/QALY.

Our study has several limitations. First, our analysis focused on the Dutch healthcare system with regard to costs. Costs for drug acquisition and administration and healthcare resource use as well as costs incurring outside the healthcare sector may be different in other countries. This might hamper the generalizability of the presented results. Moreover, the survival data from the KEYNOTE-426 trial underpinning our analysis were bound to uncertainty. Given the limited follow-up period of 42.8 months (28), long-term survival data was not available. Due to their inherent uncertainty, the selected extrapolated survival curves, despite providing relatively conservative estimates on survival, might not fully reflect the actual outcome in a real-life setting. This is also reflected in the relatively large share of patients projected to survive for at least 5 years (33.7% with pembrolizumab+axitinib and 25.6% with sunitinib compared to 11% based on real-world data from the Netherlands (8)). The application of different modelling and extrapolation methods and techniques might lead to a more favorable QALY increment for the combination treatment. However, it cannot be estimated at this point how it would also affect costs. Therefore, whether resolving this limitation would also result in a different conclusion cannot conclusively be assessed. Moreover, long-term survival outcomes and continuing responses following treatment with immunotherapies such as pembrolizumab have been suggested for melanoma patients (65). However, whether the same would apply to a(cc)RCC patients – especially when applying such treatment in combination with a TKI like axitinib – is subject to further research.

We assumed a “2 weeks on, 1 weeks off” treatment regime for sunitinib, which differs from the applied “4 weeks on, 2 weeks off” schedule applied in the KEYNOTE-426 trial. This might have implications on the toxicities, and thus, for AE-related treatments, as has been suggested by a small-scale trial (66). However, further research is required to obtain more robust evidence on this, as no comparative RCT has been conducted on this specific research question.

Data on HRQoL was used from other sources (31, 32). While the utilized data are not fully transferable to the pembrolizumab+axitinib treatment nor to the patient population in the clinical trial, we think that the results in the study by De Groot et al. provided a solid basis for estimating Dutch aRCC patients’ HRQoL in this analysis as it evaluated treatment of aRCC patients in the Netherlands (31). Further, higher HRQoL values for the pembrolizumab+axitinib arm were accounted for within the scenario analysis. The usage of the of the HRQoL data published by Bedke et al. within the KEYNOTE-426 trial (67) was decided against for the following reasons: first, the EQ-5D values for both treatment arms were elicited by applying a visual analogue scale (VAS). This contrasts the approach in the Netherlands of using the time trade-off method as a basis for estimating the HRQoL values based on the EQ-5D instrument (68). This diminishes the viability of the utility values from the referenced study for our analysis. Secondly, since the elicited QoL values were higher for sunitinib than for pembrolizumab+axitinib; applying them to this analysis would decrease the incremental effects in terms of HRQoL and, thus, of QALYs gained. This in turn would increase the ICER, further supporting our conclusion that pembrolizumab+axitinib is not cost-effective compared to sunitinib for the indication at hand (31).

Further uncertainty is spurred by insufficient data on productivity loss, especially on presenteeism and on unpaid work. The lack of data impeded a full stratification of these costs for both treatment arms. However, given that only 20% of patients in the PFS state were assumed to work, as well as the assumption that all patients have retired upon entering the PD state, we consider the general impact on the costs to be minimal. While further research exploring these aspects of productivity loss might fill this gap and provide a more robust basis for economic evaluations from a societal perspective, the effect on the actual costs incurred by presenteeism in connection with accRCC and other metastatic cancers might be modest.

For the BIA, the used patient data is an important limitation. These data, even when taking the potential range of patient numbers into account, might not be fully applicable to the future. Moreover, the analysis did not account for patients switching from sunitinib to pembrolizumab+axitinib due to e.g., a lack of response, since there was no data from real-life research available. Nonetheless, we believe that the results of our analysis provide a solid estimate on how the healthcare budget would be impacted if pembrolizumab+axitinib replaced sunitinib as the standard treatment for accRCC.

To conclude, pembrolizumab+axitinib was found not to be cost-effective as a first-line treatment for patients in the Netherlands with accRCC compared to sunitinib at a WTP threshold of €80,000/QALY from a societal perspective. Combining a substantial price reduction of pembrolizumab and axitinib (by >50%) and a shortened treatment duration may lead to a more favorable ICER. The budget impact over three years of introducing the combination treatment to the market amounts to €417.3 million upon instantaneous and full replacement of sunitinib, and to €214.9 million with increasing market penetration up to a market share of 80% in the third year.

## Data availability statement

The original contributions presented in the study are included in the article/**Supplementary Material**. Further inquiries can be directed to the corresponding author.

## Author contributions

Conceptualization and methodology: NX and CU-G. Data collection and writing of the original draft: NX. Clinical input and validation of clinical/medical data: EF. All authors contributed to the article and approved the submitted version.
