# Supplementary material for: Cost-effectiveness and budget impact of pembrolizumab+axitinib versus sunitinib in patients with advanced clear-cell renal cell carcinoma in the Netherlands
Source: Front Oncol. 2023 Jun 28;13:1205700. doi: 10.3389/fonc.2023.1205700 (PMC10336227; doi:10.3389/fonc.2023.1205700)
Supplement: Supplementary file 1 [file DataSheet_1.docx]

# Supplementary material

## Supplementary tables

Supplementary Table 1: Model input parameters (baseline values, ranges, distributions for sensitivity analysis)

| **Variable** | **Baseline value (95% CI)** | **Reference** | **Distribution** |
| --- | --- | --- | --- |
| *Incidences of adverse events – P+A group* | | | |
| Diarrhoea | 10.723% (7.795%–13.650%) | (1) | beta |
| Hypertension | 22.145% (18.215%–26.074%) | (1) | beta |
| Fatigue | 2.797% (1.237%–4.358%) | (1) | beta |
| Hand-foot syndrome (PPE) | 5.361% (3.230%–7.493%) | (1) | beta |
| ALT increase | 12.587% (9.448%–15.726%) | (1) | beta |
| AST increase | 6.760% (4.384%–9.136%) | (1) | beta |
| Thrombocytopenia | 0.000% | (1) |  |
| Neutropenia | 0.466% (~0%–1.111%) | (1) | beta |
| *Incidences of AE – sunitinib group* | | | |
| Diarrhoea | 5.412% (3.261%–7.563%) | (1) | beta |
| Hypertension | 19.765% (15.979%–23.551%) | (1) | beta |
| Fatigue | 5.412% (3.261%–7.563%) | (1) | beta |
| Hand-foot syndrome (PPE) | 4.706% (2.693%–6.719%) | (1) | beta |
| ALT increase | 2.588% (1.079%–4.098%) | (1) | beta |
| AST increase | 1.647% (0.437%–2.857%) | (1) | beta |
| Thrombocytopenia | 5.176% (3.070%–7.283%) | (1) | beta |
| Neutropenia | 7.059% (4.624%–9.494%) | (1) | beta |
| *Treatment-based health state utility parameters and dis-utilities of grade 3+ AE* | | | |
| Baseline utility | 0.760 (0.700–0.820) | (2) | beta |
| Utility PFS P+A | 0.760 (0.710–0.810) | (2) | beta |
| Utility PFS sunitinib | 0.720 (0.649–0.791) | (3) | beta |
| Utility PD | 0.660 (0.547–0.773) | (2) | beta |
| Dis-utility diarrhoea | -0.261 (-0.313–-0.209) | (4) | beta |
| Dis-utility hypertension | -0.153 (-0.201–-0.105) | (4) | beta |
| Dis-utility fatigue | -0.204 (-0.252–-0.156) | (4) | beta |
| Dis-utility hand-foot syndrome (PPE) | -0.326 (-0.381–-0.271) | (4) | beta |
| Dis-utility ALT increase | -0.150 (-0.121–-0.179) | (5) | beta |
| Dis-utility AST increase | -0.150 (-0.121–-0.179) | (5) | beta |
| Dis-utility thrombocytopenia | -0.119 (-0.154–-0.084) | (6,7) | beta |
| Dis-utility neutropenia | -0.090 (-0.120–-0.059) | (7) | beta |
| *Input parameters for AE treatment costs in PFS (indexed to 2021)* | | | |
| Treatment costs diarrhoea | €2,005.08 (€1,219.09–€2,791.07) | (8) | gamma |
| Treatment costs hypertension | €1,067.13 (€648.81–€1,485.44) | (8) | gamma |
| Treatment costs fatigue | €795.79 (€483.84–€1,107.74) | (8) | gamma |
| Treatment costs hand-foot syndrome (PPE) | €1,618.77 (€984.21–€2,253.33) | (8) | gamma |
| Treatment costs ALT increase | €314.64 (€203.68–€466.33) | (9) | gamma |
| Treatment costs AST increase | €344.83 (€223.22–€511.06) | (9) | gamma |
| Treatment costs thrombocytopenia | €3,803.54 (€3,047.58–€4,257.58) | (10) | gamma |
| Treatment costs neutropenia | €1,443.32 (€772.40–€1,999.68) | (10) | gamma |
| *Cost parameters for drug acquisition in PFS and treatment administration* | | | |
| Price of pembrolizumab (50 mg) | €1,430.28 | (11) |  |
| Price of axitinib (per 5 mg) | €68.78 | (12) |  |
| Price of sunitinib (per 50 mg) | €113.93 | (13) |  |
| Administration costs pembrolizumab | €308.98 (€248.42–€369.55) | (6,14) | gamma |
| Pharmaceutical delivery costs per pick-up of axitinib/sunitinib (indexed) | €6.62 | (15) |  |
| *Healthcare resource use/cycle parameters in PFS* | | | |
| Visit to outpatient clinic | 1.23 (0.98–1.47) | (14) | gamma |
| Inpatient care (days) | 1.74 (1.40–2.08) | (14) | gamma |
| Outpatient treatment | 0.13 (0.10–0.15) | (14) | gamma |
| Emergency treatment | 0.06 (0.05–0.08) | (14) | gamma |
| Blood test (lab) | 2.70 (2.17–3.23) | (14) | gamma |
| X-ray | 0.39 (0.31–0.46) | (14) | gamma |
| CT scan | 0.32 (0.26–0.39) | (14) | gamma |
| MRI scan | 0.06 (0.05–0.08) | (14) | gamma |
| Ultrasound | 0.13 (0.10–0.15) | (14) | gamma |
| EKG | 0.06 (0.05–0.08) | (14) | gamma |
| *Healthcare resource use cost parameters in PFS* | | | |
| Visit to outpatient clinic | €100.00 (€80.40–€119.60) | (16) | gamma |
| Inpatient care | €537.00 (€431.75–€642.25) | (16) | gamma |
| Outpatient treatment | €308.98 (€248.42–€369.55) | (16) | gamma |
| Emergency treatment | €290.26 (€233.37–€347.15) | (6) | gamma |
| Blood test (lab) | €12.48 (€10.04–€14.93) | (6) | gamma |
| X-ray | €58.26 (€46.84–€69.68) | (6) | gamma |
| CT scan | €209.11 (€168.12–€250.10) | (6) | gamma |
| MRI scan | €376.61 (€302.79–€450.42) | (6) | gamma |
| Ultrasound | €125.88 (€101.21–€150.56) | (6) | gamma |
| EKG | €50.98 (€40.99–€60.97) | (6) | gamma |
| *Patient distribution regarding second-line treatment after disease progression (first-line treatment P+A)* | | | |
| Best supportive care only | 50.00% | (17) | Dirichlet |
| Cabozantinib | 30.00% | expert opinion | Dirichlet |
| Nivolumab | 15.00% | expert opinion | Dirichlet |
| Everolimus | 1.00% | expert opinion | Dirichlet |
| Axitinib | 2.50% | expert opinion | Dirichlet |
| Sunitinib | 1.50% | expert opinion | Dirichlet |
| *Patient distribution regarding second-line treatment after disease progression (first-line treatment sunitinib)* | | | |
| Best supportive care only | 39.30% | (17) | Dirichlet |
| Cabozantinib | 12.14% | expert opinion | Dirichlet |
| Nivolumab | 48.56% | expert opinion | Dirichlet |
| Everolimus | 0.00% | expert opinion | Dirichlet |
| Axitinib | 0.00% | expert opinion | Dirichlet |
| Sunitinib | 0.00% | expert opinion | Dirichlet |
| *Drug acquisition cost parameters second-line treatment* | | | |
| Average patient bodyweight [kg] | 78.3 (62.95–93.65) | (18) | gamma |
| Cabozantinib | €213.43 | (19) | gamma |
| Nivolumab | €2,648.86 | (20) | gamma |
| Everolimus | €84.81 | (21) | gamma |
| *Healthcare resource use frequency per cycle in PD state, receiving BSC only* | | | |
| Visit to outpatient clinic | 0.903 (0.726–1.080) | (14) | gamma |
| Inpatient care (days) | 3.283 (2.640–3.926) | (14) | gamma |
| Intensive care (days) | 0.063 (0.051–0.075) | (14) | gamma |
| Outpatient treatment | 0.063 (0.051–0.075) | (14) | gamma |
| Emergency treatment | 0.126 (0.101–0.151) | (14) | gamma |
| Blood test (lab) | 0.700 (0.563–0.837) | (14) | gamma |
| X-ray | 0.518 (0.416–0.620) | (14) | gamma |
| CT scan | 0.322 (0.259–0.385) | (14) | gamma |
| MRI scan | 0.063 (0.051–0.075) | (14) | gamma |
| Ultrasound | 0.126 (0.101–0.151) | (14) | gamma |
| Skeletal scintigraphy | 0.063 (0.051–0.075) | (14) | gamma |
| EKG | 0.000 | (14) |  |
| *Healthcare resource use frequency per cycle in PD state, receiving second-line treatment* | | | |
| Visit to outpatient clinic | 1.204 (0.968–1.440) | (14) | gamma |
| Inpatient care (per day) | 1.386 (1.114–1.658) | (14) | gamma |
| Intensive care (per day) | 0.000 | (14) | gamma |
| Outpatient treatment | 0.196 (0.158–0.234) | (14) | gamma |
| Emergency treatment | 0.105 (0.084–0.126) | (14) | gamma |
| Blood test (lab) | 2.226 (1.790–2.662) | (14) | gamma |
| X-ray | 0.434 (0.349–0.519) | (14) | gamma |
| CT scan | 0.294 (0.236–0.352) | (14) | gamma |
| MRI scan | 0.000 | (14) |  |
| Ultrasound | 0.063 (0.051–0.075) | (14) | gamma |
| Skeletal scintigraphy | 0.000 | (14) |  |
| EKG | 0.035 (0.028-0.042) | (14) | gamma |
| *Additional healthcare resource use cost parameters in PD state* | | | |
| Intensive care (per day) | €2,559.75 (€2,308.89–€2,810.61) | (22) | gamma |
| Skeletal scintigraphy | €266.33 (€214.13–€318.53) | (6) | gamma |
| *Healthcare resource use for end-of-life cycle* | | | |
| Visit to outpatient clinic | 1.9 (1.528–2.272) | (6) | gamma |
| Inpatient care (days) | 18.4 (14.794–22.006) | (6) | gamma |
| Intensive care (days) | 1.2 (0.965–1.435) | (6) | gamma |
| Outpatient treatment | 0.1 (0.080–0.120) | (6) | gamma |
| Emergency treatment | 0.8 (0.643–0.957) | (6) | gamma |
| Blood test (lab) | 29 (23.316–34.684) | (6) | gamma |
| X-ray | 4.1 (3.296–4.904) | (6) | gamma |
| CT scan | 1.6 (1.286–1.914) | (6) | gamma |
| MRI scan | 0.2 (0.161–0.239) | (6) | gamma |
| Ultrasound | 0.9 (0.724–1.076) | (6) | gamma |
| Skeletal scintigraphy | 0.1 (0.080–0.120) | (6) | gamma |
| Heart scintigraphy | 0.1 (0.080–0.120) | (6) | gamma |
| EKG | 0.2 (0.161–0.239) | (6) | gamma |
| *Additional healthcare resource use cost parameters for end-of-life cycle* | | | |
| Heart scintigraphy | €20.81 (€16.73–€24.89) | (6) | gamma |
| *Cost parameters for travel and informal care* | | | |
| Cost per km travelled | €0.30 | (22) | gamma |
| Parking costs (indexed) | €3.33 (€2.03–€4.64) | (15) | gamma |
| Distance to hospital [km] | 7.0 (4.26–9.74) | (15) | gamma |
| Distance to pharmacy [km] | 1.3 (0.79–1.81) | (15) | gamma |
| Informal care costs per hour (indexed) | €15.55 (€12.50–€18.60) | (15) | gamma |
| Informal care provided P+A arm, PFS [hours/week] | 6 (3.650–8.352) | (6,14) | gamma |
| Informal care provided sunitinib arm, PFS [hours/week] | 8 (4.864–11.136) | (6,14) | gamma |
| Informal care provided P+A arm, PD [hours/week] | 10 (6.080–13.920) | (6,14) | gamma |
| Informal care provided sunitinib arm, PD [hours/week] | 10 (6.080–13.920) | (6,14) | gamma |
| *Input parameters regarding productivity loss* | | | |
| Friction period [days] | 138.439 | (15,23,24) | gamma |
| Friction period [weeks] | 19.777 | (15,23,24) | gamma |
| Productivity cost per hour | €37.30 (€22.68–€51.92) | (23) | gamma |
| Average working hours per week | 32.1 (19.517–44.683) | (25) | gamma |
| Share of working patients in the PFS state | 20% (12.160%–27.840%) | (6,14,23) | gamma |
| Share of working patients in the PD state | 0% | (6,14,23) |  |
| Hours of unpaid work lost due to treatment administration at hospital | 0.38 (0.104–0.656) | (26) | gamma |
| Hours of unpaid work lost due to treatment administration at home | 0.41 (~0–0.843) | (26) | gamma |
| Cost unpaid work per hour [indexed] | €15.55 (€9.45–€21.64) | (15) | gamma |
| Share of working time lost by informal caregiver (absenteeism) | 9.63% (2.182%–17.078%) | (27) | gamma |
| Share of working time lost by informal caregiver (presenteeism) | 15.41% (9.328%–21.492%) | (27) | gamma |

Abbreviations: AE, adverse event; ALT, alanine aminotransferase; AST, aspartate aminotransferase; BSC, best supportive care; CI: confidence interval; CT: computer tomography; EKG: electrocardiogram; MRI: magnet resonance imaging; P+A, pembrolizumab+axitinib; PD, progressed disease; PFS, progression-free survival; PPE, palmar-plantar erythrodysesthesia. Costs are indexed to 2021.

Supplementary Table 2: Extrapolation parameters and background mortality coefficients

| Parameters | Pembrolizumab+axitinib | Sunitinib |
| --- | --- | --- |
| *PFS – Lognormal* | | |
| AIC score | 1992.0567 | 1886.8204 |
| Scale (λ) | 2.7518 | 2.2825 |
| Shape (γ) | 1.2930 | 1.2139 |
| *OS – Weibull* | | |
| AIC score | 1371.6395 | 1574.8876 |
| Scale (λ) | 0.0049 | 0.0134 |
| Shape (γ) | 1.3130 | 1.2279 |
| *Background mortality (during the first year)* | | |
| Median age [years] | 62 | 61 |
| Rate of death overall | 0.0068117 | 0.0061974 |
| Rate of death w/o ccRCC | 0.006654563 | 0.006041158 |

Abbreviations: AIC, Akaike information criterion; ccRCC, clear-cell renal cell carcinoma; OS, overall survival; PFS, progression-free survival;; w/o, without.

Supplementary Table 3: Input parameters for partitioned survival model

| Parameter(s) | Standard error | Reference  (if SE given) | Reason (if SE not given) |
| --- | --- | --- | --- |
| Utility at baseline, with P+A treatment in PFS, and in PD | Derived using $SE=SD/\sqrt{n}$ | (2) |  |
| Utility with sunitinib treatment in PFS | 0.05 × µ |  | Orientation on SE of other utility parameters |
| Incidence AE P+A | derived using $se\left( p \right)=\sqrt{p*(1-p)/n}$ | (1) |  |
| Incidence of thrombocytopenia with P+A | none |  | Prevalence of 0%, therefore no uncertainty assumed |
| Incidence AE sunitinib | derived using $se\left( p \right)=\sqrt{p*(1-p)/n}$ | (1) |  |
| Dis-utilities diarrhoea, hypertension, fatigue, PPE | given (derived from range using 1.96 × SE) |  |  |
| Dis-utilities ALT/AST increase | 0.1 × µ |  | Based on assumption in literature (28); factor choice based on SE for disutilities diarrhoea, hypertension, fatigue, PPE |
| Dis-utility neutropenia, | given | (7) |  |
| Dis-utility thrombocytopenia | 0.15 × µ |  | Orientation on SE of disutilities decreased platelet count, neutropenia, decreased neutrophil count |
| Cost inflation indices | none |  | Index values are fixed, no uncertainty |
| Unit costs for treatment of diarrhoea, hypertension, fatigue, PPE, ALT/AST increase | 0.2 × µ |  | Varying degree of necessary treatment and use of healthcare resources, depending on individual afflicted patient |
| Unit costs for treatment of thrombocytopenia, neutropenia | Derived using $SE=SD/\sqrt{n}$ | (10) |  |
| Unit costs pembrolizumab, axitinib, sunitinib | none |  | Prices assumed as fixed |
| Unit dosages pembrolizumab, axitinib, sunitinib | none |  | Predetermined value, no uncertainty |
| Patient dosages pembrolizumab, axitinib, sunitinib; number of vials needed for 1 administration of pembrolizumab | none |  | Based on mandated treatment regimen in clinical trial, no uncertainty |
| Daily administrations for axitinib / sunitinib | none |  | Mandated treatment regimen in clinical trial, no uncertainty |
| Days per cycle; number of administrations of pembrolizumab / axitinib / sunitinib per cycle | none |  | Based on mandated treatment regimen in clinical trial, no uncertainty |
| (unindexed) administration costs for chemotherapy and pharmacy delivery costs | none |  | Prices assumed as fixed |
| Pack sizes axitinib / sunitinib | none |  | Tablet packs assumed to always contain the denoted number of tablets |
| Average price for outpatient clinic visit (1–2 days), inpatient care (max. 5 days), and day care treatment (with *zorgproductcode*) | 0.1 × µ |  | Based on autonomous negotiability between provider and insurer/payer; coincides with ZIN practice (29) |
| Avgerage unit costs for emergency treatment and monitoring tests (unindexed) | 0.1 × µ |  | Based on autonomous negotiability between provider and insurer/payer; coincides with ZIN practice (29) |
| Frequency of healthcare resource use per month in PFS | 0.1 × µ |  | Accounts for variety in healthcare resource use per patient based on individual characteristics; coincides with ZIN practice (29) |
| Scaling parameters (month to cycle / to 1 visit in outpatient clinic / to 1 day in inpatient care) | none |  | Calculation based on provided data on corresponding healthcare resources |
| Patient distribution for BSC / second-line treatment after progressing with pembrolizumab + axitinib | derived using $se\left( p \right)=\sqrt{p*(1-p)/n}$ | (17) |  |
| Patient distributions within second-line treatment after progressing with pembrolizumab + axitinib | Derived using Dirichlet distribution |  |  |
| Patient distribution for BSC / second-line treatment after progressing sunitinib | derived using $se\left( p \right)=\sqrt{p*(1-p)/n}$ | (17) |  |
| Patient distributions within second-line treatment after progressing with sunitinib | Derived using Dirichlet distribution |  |  |
| Patient distribution sunitinib as second-line treatment after progressing with sunitinib | none |  | Prevalence of 0%, therefore no uncertainty |
| Duration of second-line treatment (cabozantinib / nivolumab / everolimus / axitinib / sunitinib) | 0.2 × µ |  | Accounts for variety of treatment duration based on individual patient and the characteristics of their disease and constitution |
| Bodyweight of patient | 0.1 × µ |  | Accounts for variety regarding bodyweight among patients |
| Unit dosage and vial content of nivolumab | none |  | Mandated treatment regimen / deviations in the manufacturing process not considered substantial to cause uncertainty |
| Number days in a treatment cycle of nivolumab | none |  | Mandated treatment regimen, therefore no uncertainty |
| Unit dosages for cabozantinib / everolimus/ axitinib / sunitinib | none |  | Predetermined value, no uncertainty |
| Scaling parameter for days in which sunitinib is administered | none |  | Mandated treatment regimen (administered during the first 4 weeks within a 6-week cycle, therefore no uncertainty |
| Unit costs for cabozantinib / nivolumab / everolimus/ axitinib / sunitinib | none |  | Prices assumed as fixed |
| Patient dosages for cabozantinib / nivolumab / everolimus/ axitinib / sunitinib | none |  | Mandated treatment regimen, thus no uncertainty |
| Pack sizes of cabozantinib / everolimus / axitinib / sunitinib | none |  | Tablet packs assumed to always contain the denoted number of tablets |
| Unit costs of 1 day in ICU / 1 skeletal scintigraphy | 0.05 × µ |  | Based on autonomous negotiability between provider and insurer/payer; coincides with ZIN practice (29) |
| Frequency of healthcare resource use per month in PD, while receiving BSC only | 0.1 × µ |  | Accounts for variety in healthcare resource use per patient based on individual characteristics; coincides with ZIN practice (29) |
| Frequency of healthcare resource use per month in PD, while receiving second-line chemotherapy | 0.1 × µ |  | Accounts for variety in healthcare resource use per patient based on individual characteristics; coincides with ZIN practice (29) |
| BSC costs | 0.2 × µ |  | Accounts for variety of BSC provided on an individual level (30) |
| Unit costs for heart scintigraphy | 0.1 × µ |  | Based on autonomous negotiability between provider and insurer/payer; coincides with ZIN practice (29) |
| Frequency of healthcare resource use in the last month of life | 0.1 × µ |  | Accounts for variety in healthcare resource use per patient based on individual characteristics; coincides with ZIN practice (29) |
| Distance to hospital / pharmacy / GP | 0.2 × µ |  | Accounts for infrastructural differences (urban/rural areas) where patient’s household is located |
| Unit cost per kilometre travelled | none |  | Maximum tariff provided by NZa; no uncertainty assumed |
| Unit cost for parking | 0.2 × µ |  | Accounts for differences in parking fees and parking time |
| Parameter for two-way travel | none |  | Predetermined value; no uncertainty |
| Parameter for two two-way travels | none |  | Predetermined value; no uncertainty |
| Travel frequencies for treatment administration (pembrolizumab) or getting a new pack at the pharmacy (axitinib / sunitinib) | none |  | Linked to mandated treatment regimen and pack size, therefore no uncertainty |
| Travel frequency regarding inpatient care in PFS | none |  | Assumed at 1 per cycle (one hospital visit covering all inpatient care days per cycle) |
| Travel frequency regarding inpatient care in PD, while receiving BSC only | none |  | Assumed at 1 per cycle (one hospital visit covering all inpatient care days per cycle) |
| Travel frequency regarding inpatient care in PD, while receiving second-line treatment | none |  | Assumed at 1 per cycle (one hospital visit covering all inpatient care days per cycle) |
| Hourly rate for informal care (unindexed) | 0.1 × µ |  | Accounts for variety in paid hourly rate of informal care |
| Hours of informal care provided per week in PFS state (P+A/sunitinib) | 0.2 × µ |  | Accounts for varying hours of informal care provided based on individual patient characteristics |
| Hours of informal care provided per week in PD state (P+A/sunitinib) | 0.2 × µ |  | Accounts for varying hours of informal care provided based on individual patient characteristics |
| Scaling parameter: weeks in a cycle | none |  | Predetermined value; no uncertainty |
| Input parameters to calculate friction period | none |  | Predetermined values in equation; data provided by CBS (23,24) not assumed to be bound to uncertainty |
| Productivity costs per hour | 0.2 × µ |  | Accounts for the variety of professions a patient can pursue |
| Working hours per week | 0.2 × µ |  | Accounts for the variety in working hours/fulltime equivalents regarding a patient’s job |
| Working hours per week in PD | none |  | Assumed at 0 |
| Share of patients working in PFS | 0.2 × µ |  | Accounts for individual patient characteristics and their ability to still work with aRCC |
| Share of patients working in PD | none |  | Assumed at 0% |
| Productivity loss regarding unpaid work per administration of drug at home / at the hospital | Derived using $SE=SD/\sqrt{n}$ | (26) |  |
| Productivity cost for unpaid work | 0.2 × µ |  | Accounts for the variety of unpaid work pursued |
| Working time lost by informal caregivers (absenteeism / presenteeism) | Derived using $SE=SD/\sqrt{n}$ | (27) |  |

Abbreviations and symbols: AE, adverse events; ALT, alanine aminotransferase; aRCC, advanced renal cell carcinoma; AST, aspartate aminotransferase; BSC: best supportive care; GP, general practitioner; ICU, intensive care unit; P+A, pembrolizumab+axitinib; PD, progressed disease state; PFS, progression-free survival; PPE, palmar-plantar erythrodysesthesia; SD, standard deviation; SE, standard error; µ, mean.

Supplementary Table 4: Background mortality calculations

| **Rate of death** | | | | | | | | | | | | | |
| --- | --- | --- | --- | --- | --- | --- | --- | --- | --- | --- | --- | --- | --- |
| **Age (years)** | **Living overall** | | | **Deceased overall** | | | **IMR overall** | | | **RoD overall** | **Deceased w/o ccRCC** | **IMR w/o ccRCC** | **ROD w/o ccRCC** |
| 60 | 94285 | | | 505 | | | 0.005356101 | | | 0.0053418 | 490.258 | 0.005199745 | 0.00518625 |
| 61 | 93780 | | | 583 | | | 0.006216677 | | | 0.0061974 | 568.258 | 0.00605948 | 0.006041158 |
| 62 | 93197 | | | 637 | | | 0.006834984 | | | 0.0068117 | 622.258 | 0.006676803 | 0.006654563 |
| 63 | 92560 | | | 693 | | | 0.007487035 | | | 0.0074591 | 678.258 | 0.007327766 | 0.007300983 |
| 64 | 91867 | | | 806 | | | 0.008773553 | | | 0.0087352 | 791.258 | 0.008613082 | 0.008576096 |
| 65 | 91061 | | | 833 | | | 0.009147714 | | | 0.0091060 | 818.258 | 0.008985823 | 0.008945571 |
| 66 | 90228 | | | 928 | | | 0.010285056 | | | 0.0102323 | 913.258 | 0.01012167 | 0.010070618 |
| 67 | 89300 | | | 1003 | | | 0.011231803 | | | 0.0111690 | 988.258 | 0.011066719 | 0.011005708 |
| 68 | 88297 | | | 1098 | | | 0.012435304 | | | 0.0123583 | 1083.258 | 0.012268344 | 0.012193395 |
| 69 | 87199 | | | 1192 | | | 0.013669882 | | | 0.0135769 | 1177.258 | 0.01350082 | 0.013410093 |
| 70 | 86007 | | | 1248 | | | 0.014510447 | | | 0.0144057 | 1233.258 | 0.014339042 | 0.014236728 |
| 71 | 84759 | | | 1420 | | | 0.016753383 | | | 0.0166138 | 1405.258 | 0.016579455 | 0.016442772 |
| 72 | 83339 | | | 1529 | | | 0.018346752 | | | 0.0181795 | 1514.258 | 0.01816986 | 0.018005784 |
| 73 | 81810 | | | 1649 | | | 0.02015646 | | | 0.0199547 | 1634.258 | 0.019976262 | 0.019778059 |
| 74 | 80161 | | | 1741 | | | 0.021718791 | | | 0.0214846 | 1726.258 | 0.021534886 | 0.021304666 |
| 75 | 78420 | | | 2044 | | | 0.026064779 | | | 0.0257280 | 2024.302 | 0.025813593 | 0.025483271 |
| 76 | 76376 | | | 2238 | | | 0.029302399 | | | 0.0288772 | 2218.302 | 0.02904449 | 0.028626753 |
| 77 | 74138 | | | 2369 | | | 0.031953924 | | | 0.0314488 | 2349.302 | 0.03168823 | 0.03119142 |
| 78 | 71769 | | | 2615 | | | 0.036436344 | | | 0.0357805 | 2595.302 | 0.03616188 | 0.03551585 |
| 79 | 69154 | | | 2841 | | | 0.041082222 | | | 0.0402498 | 2821.302 | 0.04079738 | 0.03997637 |
| 80 | 66313 | | | 3002 | | | 0.045270158 | | | 0.0442608 | 2982.302 | 0.044973112 | 0.043976813 |
| 81 | 63311 | | | 3291 | | | 0.051981488 | | | 0.0506536 | 3271.302 | 0.051670357 | 0.050358142 |
| 82 | 60020 | | | 3437 | | | 0.057264245 | | | 0.0556555 | 3417.302 | 0.056936055 | 0.055345526 |
| 83 | 56583 | | | 3750 | | | 0.066274323 | | | 0.0641259 | 3730.302 | 0.065926197 | 0.063800044 |
| *Source* | (31) | | | (31) | | |  | | |  |  |  |  |
| **Mortality accRCC (2020)** | | | | | | | |  |  |  |  |  |  |
| **Age group** | | | **Deceased overall** | | | **Deceased ccRCC** | |  |  |  |  |  |  |
| 60-74 years | | | 351.00 (32) | | | 221.130 | |  |  |  |  |  |  |
| 75+ years | | | 469.00 (32) | | | 295.470 | |  |  |  |  |  |  |
| mean per LY (60-74) | | | 23.40 (32) | | | 14.742 | |  |  |  |  |  |  |
| mean per LY (75+) | | | 31.27 (32) | | | 19.698 | |  |  |  |  |  |  |
| *Scaling factor to 1 LY* | | | *15.0* | | |  | |  |  |  |  |  |  |
| **Extrapolation factors** | | | | | | | |  |  |  |  |  |  |
| **KC subtypes** | | **Ratio** | | | **Source** | | |  |  |  |  |  |  |
| Ratio RCC to KC | | 90% | | | (33) | | |  |  |  |  |  |  |
| Ratio ccRCC to RCC | | 70% | | | (34) | | | |  |  |  |  |  |
| Ratio ccRCC to KC | | 63% | | | extrapolated | | |  |  |  |  |  |  |

Abbreviations: accRCC, advanced clear-cell renal cell carcinoma; ccRCC, clear-cell renal cell carcinoma; IMR, instantaneous mortality rate; KC, kidney cancer; LY, life year; RCC, renal cell carcinoma; RoD: rate of death; w/o, without.

# References

1. Powles T, Plimack ER, Soulières D, Waddell T, Stus V, Gafanov R, Nosov D, Pouliot F, Melichar B, Vynnychenko I, et al. Pembrolizumab plus axitinib versus sunitinib monotherapy as first-line treatment of advanced renal cell carcinoma (KEYNOTE-426): extended follow-up from a randomised, open-label, phase 3 trial. *Lancet Oncol* (2020) 21:1563–1573. doi: 10.1016/S1470-2045(20)30436-8

2. de Groot S, Redekop WK, Versteegh MM, Sleijfer S, Oosterwijk E, Kiemeney LALM, Uyl-de Groot CA. Health-related quality of life and its determinants in patients with metastatic renal cell carcinoma. *Quality of Life Research* (2018) 27:115–124. doi: 10.1007/s11136-017-1704-4

3. Cella D, Michaelson MD, Bushmakin AG, Cappelleri JC, Charbonneau C, Kim ST, Li JZ, Motzer RJ. Health-related quality of life in patients with metastatic renal cell carcinoma treated with sunitinib vs interferon-α in a phase III trial: Final results and geographical analysis. *Br J Cancer* (2010) 102:658–664. doi: 10.1038/sj.bjc.6605552

4. Swinburn P, Lloyd A, Nathan P, Choueiri TK, Cella D, Neary MP. Elicitation of health state utilities in metastatic renal cell carcinoma. *Curr Med Res Opin* (2010) 26:1091–1096. doi: 10.1185/03007991003712258

5. Simons CL, Malone D, Wang M, Maglinte GA, Inocencio T, Wade SW, Bennison C, Shah B. Cost-effectiveness for KTE-X19 CAR T therapy for adult patients with relapsed/refractory mantle cell lymphoma in the United States. *J Med Econ* (2021) 24:421–431. doi: 10.1080/13696998.2021.1894158

6. Zorginstituut Nederland. Farmacotherapeutisch rapport voor avelumab plus axitinib (Bavencio® en Inlyta®) bij de eerstelijnsbehandeling van volwassen patiënten met gevorderd niercelcarcinoom. (2020). www.zorginstituutnederland.nl [Accessed January 23, 2023]

7. Nafees B, Stafford M, Gavriel S, Bhalla S, Watkins J. Health state utilities for non small cell lung cancer. *Health Qual Life Outcomes* (2008) 6:84. doi: 10.1186/1477-7525-6-84

8. Mickisch G, Gore M, Escudier B, Procopio G, Walzer S, Nuijten M. Costs of managing adverse events in the treatment of first-line metastatic renal cell carcinoma: Bevacizumab in combination with interferon-α2a compared with sunitinib. *Br J Cancer* (2010) 102:80–86. doi: 10.1038/sj.bjc.6605417

9. Campone M, Yang H, Faust E, Kageleiry A, Signorovitch JE, Zhang J, Gao H. Cost of adverse events during treatment with everolimus plus exemestane or single-agent chemotherapy in patients with advanced breast cancer in Western Europe. *J Med Econ* (2014) 17:837–845. doi: 10.3111/13696998.2014.959589

10. Bouwmans C, Janssen J, Huijgens P, Uyl-De Groot C. Costs of haematological adverse events in chronic myeloid leukaemia patients: A retrospective cost analysis of the treatment of anaemia, neutropenia and thrombocytopenia in patients with chronic myeloid leukaemia. *J Med Econ* (2009) 12:164–169. doi: 10.3111/13696990903149479

11. Zorginstituut Nederland. KEYTRUDA INFUSIEPOEDER FLACON 50MG _ Medicijnkosten.nl. *Medicijnkosten.nl* (2022) https://www.medicijnkosten.nl/medicijn?artikel=KEYTRUDA+INFUSIEPOEDER+FLACON+50MG&id=54aeb7397a0bba0f05a81f7db8341ded [Accessed November 15, 2022]

12. Zorginstituut Nederland. INLYTA TABLET FILMOMHULD 5MG _ Medicijnkosten.nl. *Medicijnkosten.nl* (2022) https://www.medicijnkosten.nl/medicijn?artikel=INLYTA+TABLET+FILMOMHULD+5MG&id=a58abcc7af3969091c824ba509bcae4d [Accessed November 15, 2022]

13. Zorginstituut Nederland. SUTENT CAPSULE 50MG _ Medicijnkosten.nl. *Medicijnkosten.nl* (2022) https://www.medicijnkosten.nl/medicijn?artikel=SUTENT+CAPSULE+50MG&id=c59cbc519c458399e1cc61bbfd055e63 [Accessed November 15, 2022]

14. Zorginstituut Nederland. Farmacotherapeutisch rapport ipilimumab in combinatie met nivolumab (Yervoy® en Opdivo®) bij de eerstelijns behandeling van gevorderd niercelcarcinoom met intermediair/ongunstig risicoprofiel bij volwassenen. (2019). www.zorginstituutnederland.nl

15. Hakkaart-van Rooijen L, van der Linden N, Bouwmans C, Kanters T, Tan SS. Kostenhandleiding: Methodologie van kostenonderzoek en referentieprijzen voor economische evaluaties in de gezondheidszorg. https://www.zorginstituutnederland.nl/binaries/zinl/documenten/publicatie/2016/02/29/richtlijn -voor-het-uitvoeren-van-economische-evaluaties-in-degezondheidszorg/ Richtlijn+voor+het+uitvoeren+van+economische+evaluaties+in+de+gezond heidszorg+%28verdiepingsmodules%29.pdf [Accessed November 9, 2022]

16. Nederlandse Zorgautoriteit. DIS open data. (2022) https://opendisdata.nl/ [Accessed November 15, 2022]

17. Rini BI, Plimack ER, Stus V, Gafanov R, Hawkins R, Nosov D, Pouliot F, Alekseev B, Soulières D, Melichar B, et al. Pembrolizumab plus Axitinib versus Sunitinib for Advanced Renal-Cell Carcinoma. *New England Journal of Medicine* (2019) 380:1116–1127. doi: 10.1056/nejmoa1816714

18. Centraal Bureau voor de Statistiek. Lengte en gewicht van personen, ondergewicht en overgewicht; vanaf 1981. (2022) https://www.cbs.nl/nl-nl/cijfers/detail/81565NED?dl=35805 [Accessed December 12, 2022]

19. Zorginstituut Nederland. CABOMETYX TABLET FILMOMHULD 60MG _ Medicijnkosten.nl. *Medicijnkosten.nl* (2022) https://www.medicijnkosten.nl/medicijn?artikel=CABOMETYX+TABLET+FILMOMHULD+60MG&id=adbe93dd6cb7bd488d1153466ce3bc3d [Accessed November 15, 2022]

20. Zorginstituut Nederland. OPDIVO INFVLST CONC 10MG_ML FLACON 24ML _ Medicijnkosten.nl. *Medicijnkosten.nl* (2022) https://www.medicijnkosten.nl/medicijn?artikel=OPDIVO+INFVLST+CONC+10MG%2FML+FLACON+24ML&id=6d0a627477777b15a9cecd70abe2205f [Accessed November 15, 2022]

21. Zorginstituut Nederland. EVEROLIMUS ACCORD TABLET 10MG _ Medicijnkosten.nl. *Meddicijnkosten.nl* (2022) https://www.medicijnkosten.nl/medicijn?artikel=EVEROLIMUS+ACCORD+TABLET+10MG&id=63c3ab6486cc2d8a31b2612ce423c9e6 [Accessed November 15, 2022]

22. Nederlandse Zorgautoriteit. NZa zorgproductapplicatie. (2022) https://zorgproducten.nza.nl/ZoekZorgproduct.aspx [Accessed January 23, 2023]

23. Centraal Bureau voor de Statistiek. De arbeidsmarkt in cijfers 2021. (2022). https://www.cbs.nl/-/media/_pdf/2022/17/daic2021.pdf [Accessed November 9, 2022]

24. Centraal Bureau voor de Statistiek. Vacatures. (2022) https://www.cbs.nl/nl-nl/visualisaties/dashboard-arbeidsmarkt/vacatures [Accessed November 9, 2022]

25. Centraal Bureau voor de Statistiek. Werkzame beroepsbevolking; arbeidsduur. *StatLine* (2022) https://www.cbs.nl/nl-nl/cijfers/detail/85275NED?q=werkzame%20beroepsbevolking%20arbeidsduur [Accessed January 23, 2023]

26. Franken M, Kanters T, Coenen J, de Jong P, Jager A, Groot CU de. Hospital-based or home-based administration of oncology drugs? A micro-costing study comparing healthcare and societal costs of hospital-based and home-based subcutaneous administration of trastuzumab. *Breast* (2020) 52:71–77. doi: 10.1016/j.breast.2020.05.001

27. Mazanec SR, Daly BJ, Douglas SL, Lipson AR. Work productivity and health of informal caregivers of persons with advanced cancer. *Res Nurs Health* (2011) 34:483–495. doi: 10.1002/nur.20461

28. Simons CL, Malone D, Wang M, Maglinte GA, Inocencio T, Wade SW, Bennison C, Shah B. Cost-effectiveness for KTE-X19 CAR T therapy for adult patients with relapsed/refractory mantle cell lymphoma in the United States. *J Med Econ* (2021) 24:421–431. doi: 10.1080/13696998.2021.1894158

29. Zorginstituut Nederland. Farmacotherapeutisch rapport voor avelumab plus axitinib (Bavencio® en Inlyta®) bij de eerstelijnsbehandeling van volwassen patiënten met gevorderd niercelcarcinoom. (2020). www.zorginstituutnederland.nl

30. Uyl-de Groot CA, van Rooijen EM, Punt CJA, Pescott CP. Real-world cost-effectiveness of cetuximab in the third-line treatment of metastatic colorectal cancer based on patient chart review in the Netherlands. *Health Econ Rev* (2018) 8:13. doi: 10.1186/s13561-018-0197-3

31. Kanker.nl. Overlevingscijfers van nierkanker _ Kanker.nl. https://www.kanker.nl/kankersoorten/nierkanker/algemeen/overlevingscijfers-van-nierkanker [Accessed December 7, 2022]

32. Integraal Kankercentrum Nederland. NKR Cijfers. https://iknl.nl/nkr-cijfers?fs%7Cepidemiologie_id=526&fs%7Ctumor_id=357&fs%7Cregio_id=550&fs%7Cperiode_id=564%2C565%2C566%2C567%2C568%2C569%2C570%2C571%2C572%2C573%2C574%2C575%2C576%2C577%2C578%2C579%2C580%2C581%2C582%2C583%2C584%2C585%2C586%2C587%2C588%2C589%2C590%2C591%2C592%2C593%2C594%2C563%2C562&fs%7Cgeslacht_id=645&fs%7Cleeftijdsgroep_id=678&fs%7Cjaren_na_diagnose_id=688&fs%7Ceenheid_id=704&cs%7Ctype=line&cs%7CxAxis=periode_id&cs%7Cseries=epidemiologie_id&ts%7CrowDimensions=periode_id&ts%7CcolumnDimensions=&lang%7Clanguage=nl [Accessed November 7, 2022]

33. Motzer RJ, Agarwal N, Beard C, Bolger GB, Boston B, Carducci MA, Choueiri TK, Figlin RA, Fishman ; Mayer, Hancock SL, et al. Kidney Cancer Clinical Practice Guidelines in Oncology TM Kidney Cancer Clinical Practice Guidelines in Oncology. *Journal of the National Comprehensive Cancer Network |* (2009) 7:618–630. www.nccn.org.

34. Jonasch E, Walker CL, Rathmell WK. Clear cell renal cell carcinoma ontogeny and mechanisms of lethality. *Nat Rev Nephrol* (2021) 17:245–261. doi: 10.1038/s41581-020-00359-2
