# Supplementary figures and images for: Cost-effectiveness and budget impact of pembrolizumab+axitinib versus sunitinib in patients with advanced clear-cell renal cell carcinoma in the Netherlands
Source: Front Oncol. 2023 Jun 28;13:1205700. doi: 10.3389/fonc.2023.1205700 (PMC10336227; doi:10.3389/fonc.2023.1205700)

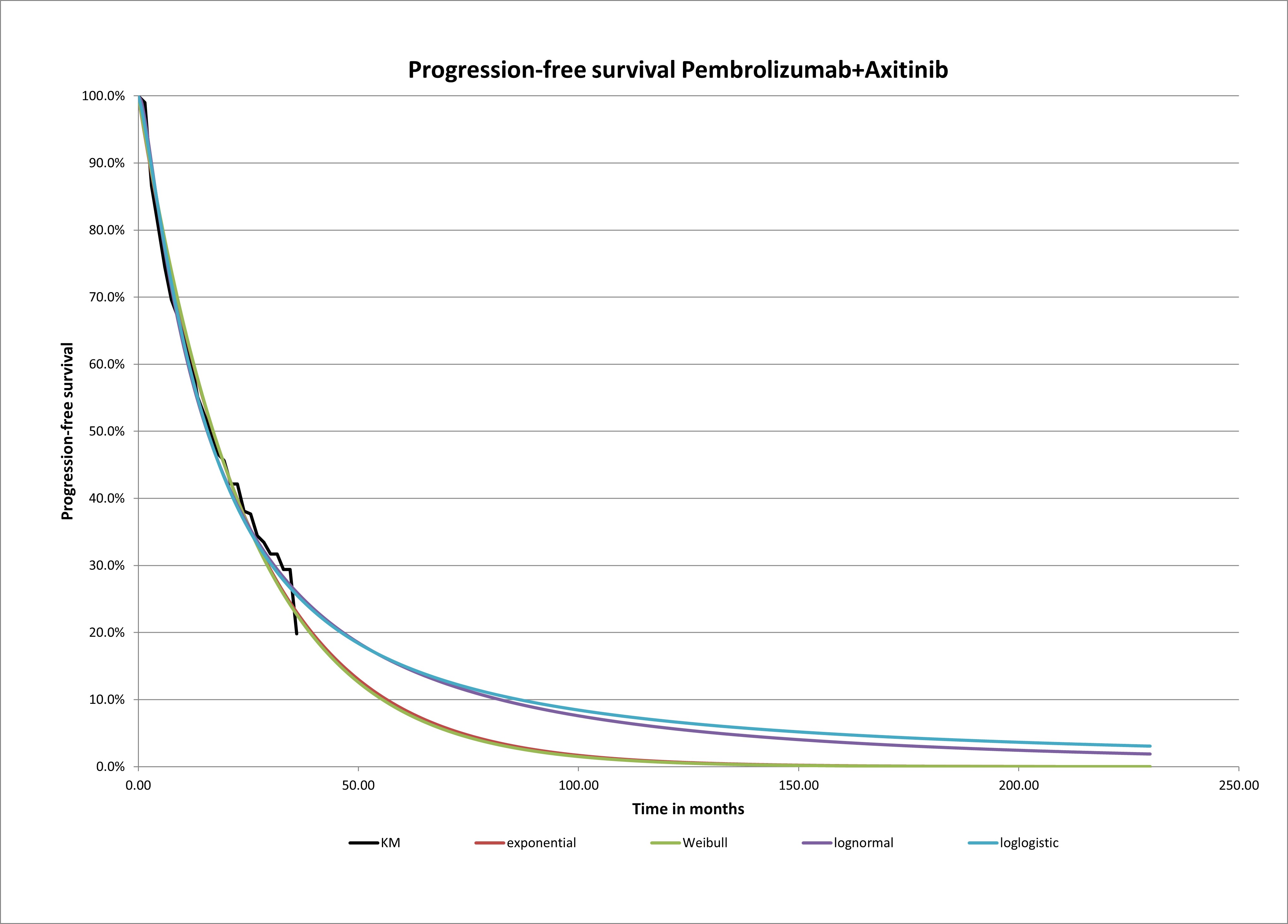

Supplement: Supplementary file 2 [file Image_1.jpeg]

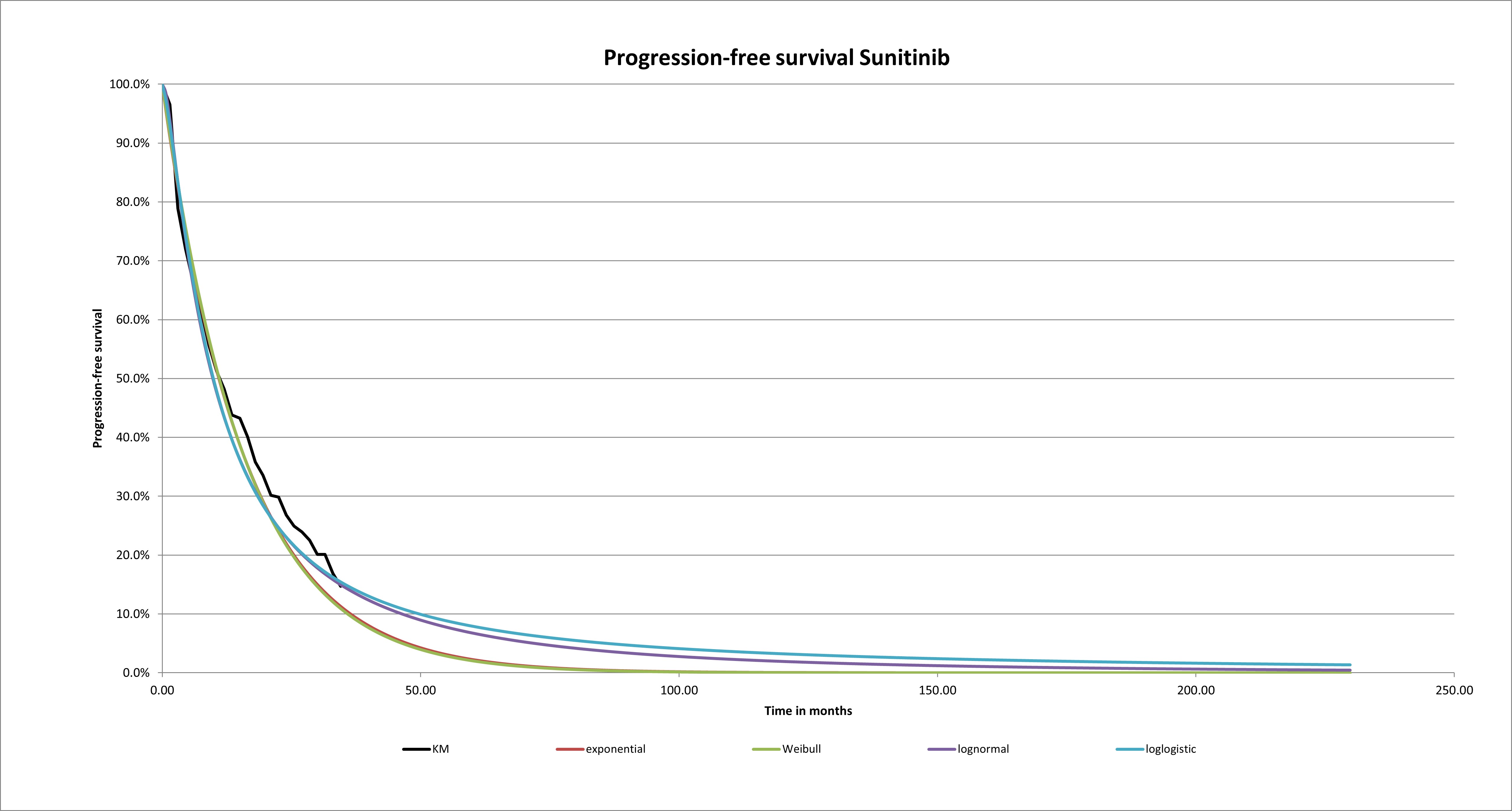

Supplement: Supplementary file 3 [file Image_2.jpeg]

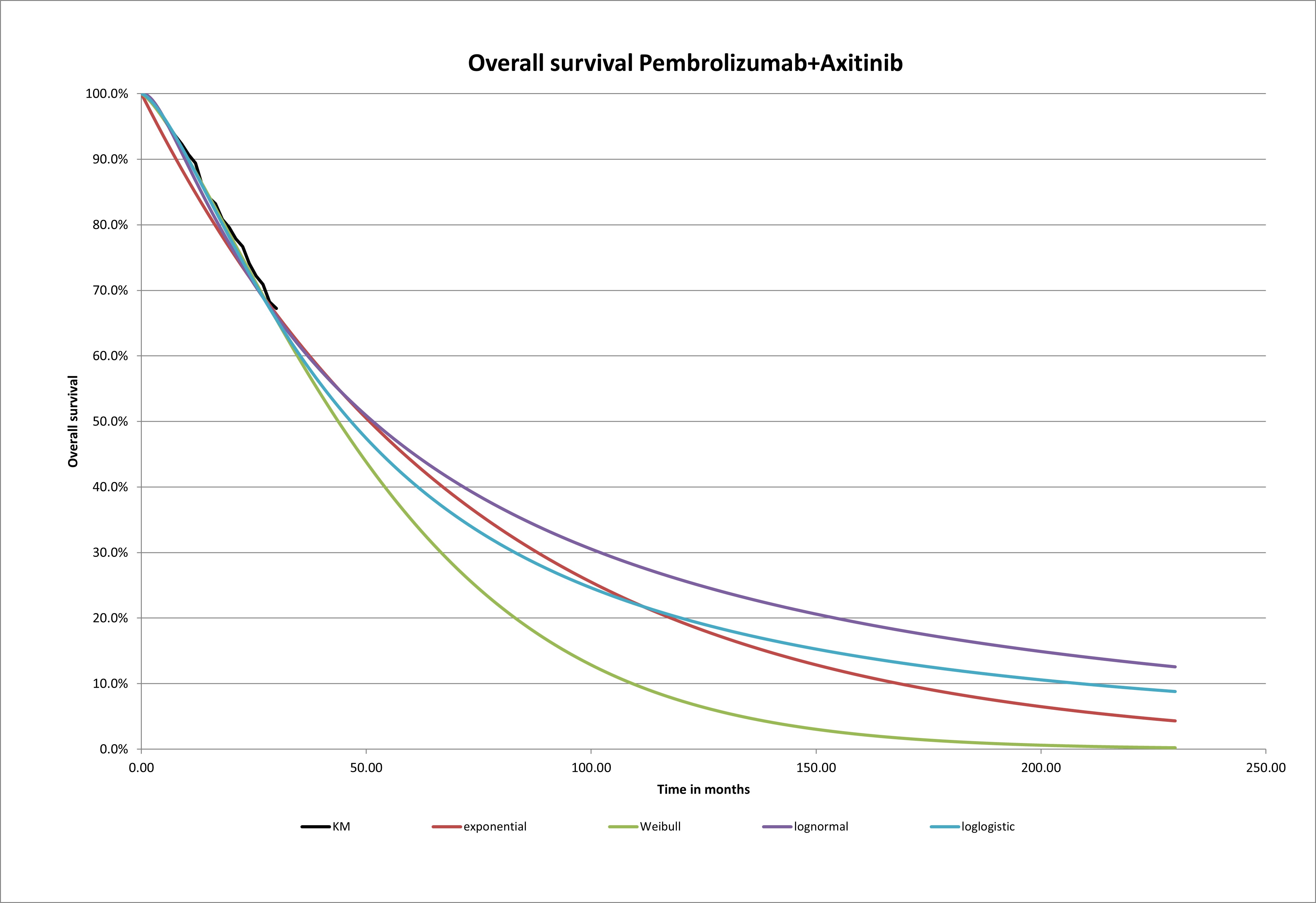

Supplement: Supplementary file 4 [file Image_3.jpeg]

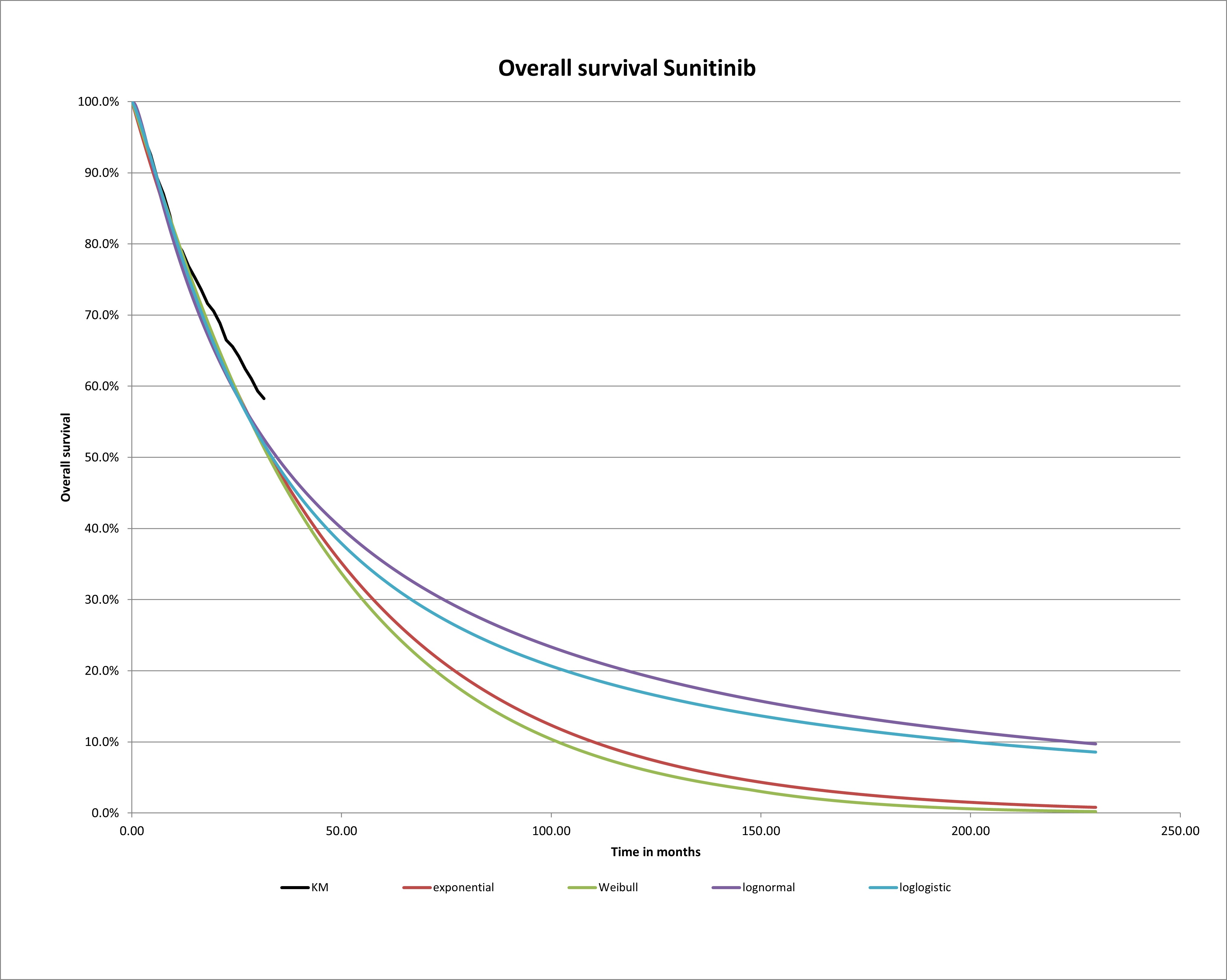

Supplement: Supplementary file 5 [file Image_4.jpeg]
